# Transcriptional synergy in human aortic endothelial cells is vulnerable to combination p300/CBP and BET bromodomain inhibition

**DOI:** 10.1016/j.isci.2024.110011

**Published:** 2024-05-16

**Authors:** Ronan C. Bracken, Lindsay M. Davison, Dennis P. Buehler, Maci E. Fulton, Emily E. Carson, Quanhu Sheng, Lindsey K. Stolze, Christelle Guillermier, Matthew L. Steinhauser, Jonathan D. Brown

**Affiliations:** 1 Department of Biochemistry, Vanderbilt University School of Medicine, Nashville, TN 37232, USA; 2 Division of Cardiovascular Medicine, Vanderbilt University Medical Center, Nashville, TN 37232, USA; 3 Department of Biostatistics, Vanderbilt University School of Medicine, Nashville, TN 37232, USA; 4 Harvard Medical School, Boston, MA 02115, USA; 5 Center for NanoImaging, Cambridge MA 02115, USA; 6 Department of Medicine, Division of Genetics, Brigham and Women’s Hospital, Boston, MA 02115, USA; 7 Aging Institute, University of Pittsburgh School of Medicine, Pittsburgh, PA 15261, USA; 8 Center for Quantitative Sciences, Vanderbilt University Medical Center, Nashville, TN 3723, USA

**Keywords:** Components of the immune system, Cell biology, Systems biology, Genomics, Transcriptomics, Chemogenomics

## Abstract

Combinatorial signaling by proinflammatory cytokines synergizes to exacerbate toxicity to cells and tissue injury during acute infections. To explore synergism at the gene-regulatory level, we investigated the dynamics of transcription and chromatin signaling in response to dual cytokines by integrating nascent RNA imaging mass spectrometry, RNA sequencing, amplification-independent mRNA quantification, assay for transposase-accessible chromatin using sequencing (ATAC-seq), and transcription factor profiling. Costimulation with interferon-gamma (IFNγ) and tumor necrosis factor alpha (TNFα) synergistically induced a small subset of genes, including the chemokines *CXCL9*, -*10*, and -*11*. Gene induction coincided with increased chromatin accessibility at non-coding regions enriched for p65 and STAT1 binding sites. To discover coactivator dependencies, we conducted a targeted chemogenomic screen of transcriptional inhibitors followed by modeling of inhibitor dose-response curves. These results identified high efficacy of either p300/CREB-binding protein (CBP) or bromodomain and extra-terminal (BET) bromodomain inhibitors to disrupt induction of synergy genes. Combination p300/CBP and BET bromodomain inhibition at half-maximal inhibitory concentrations (subIC_50_) synergistically abrogated IFNγ/TNFα-induced chemokine gene and protein levels.

## Highlights

• IFNγ/TNFα synergistically induce a subset of proinflammatory transcripts
• STAT1/p65 co-bind at enhanced accessible elements in response to IFNγ/TNFα
• Synergistic transcription is vulnerable to combined CBP/p300 and BET inhibition
• Open chromatin and STAT1/p65 binding are unaffected by CBP/p300 and BET inhibition

## Graphical abstract

**Figure:**
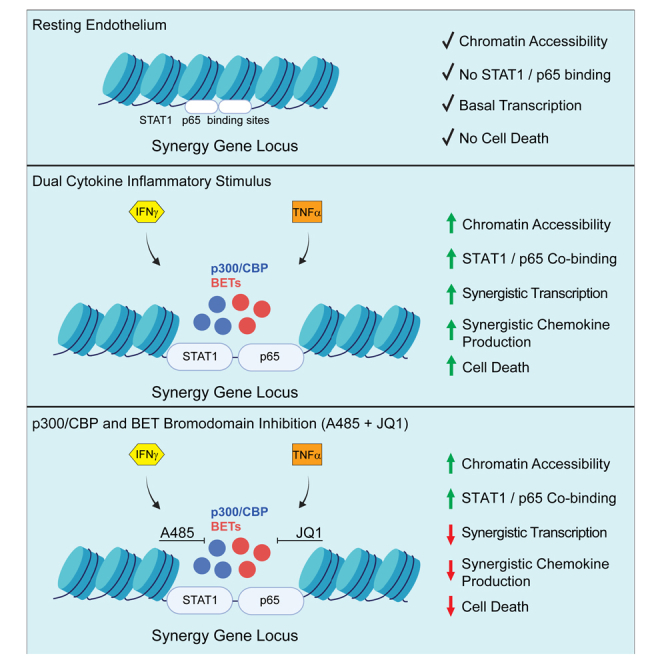

## Introduction

Acute inflammation triggered by infectious microorganisms is a tightly regulated process essential to ensure human health. As part of this response, an array of cytokines released by the innate and adaptive immune system mobilize host cellular defenses with the goal of clearing invading pathogens and resolving tissue damage. However, in a subset of infections including severe acute respiratory syndrome coronavirus 2 (SARS-CoV-2), cytokine storm develops featuring massive release of proinflammatory cytokines that promote progressive cell death, organ damage, and heightened mortality in humans.^1^ In the particular case of severe SARS-CoV-2 illness, high circulating levels of interferon-gamma (IFNγ) and tumor necrosis factor alpha (TNFα) predominate.^2^ At a cellular level, the combination of IFNγ and TNFα signaling causes PANoptosis, which if unchecked over time ultimately causes shock and death at the organism level.^2^

Gene regulation is one important facet of control in cytokine responses. Signaling elicited by IFNγ and TNFα promotes rapid transcription of proinflammatory genes by activating two master transcription factors (TFs): signal transducer and activator of transcription-1 (STAT1) and p65/nuclear factor-kappa B (NF-κB), respectively. In the absence of inflammatory stimuli, STAT1 and p65 reside as inactive proteins in the cytosol.^3^^,^^4^ IFNγ or TNFα signal transduction cascades prompt phosphorylation and rapid translocation of each TF to the nucleus where they bind unique sequence-specific regulatory DNA elements, assemble coregulatory complexes including mediator and histone remodeling factors, and alter chromatin conformation, thereby driving RNA polymerase II (Pol II)-dependent transcription of inflammatory genes.^5^

Transcriptional synergy—when induction of a gene by two or more stimuli is greater than the additive effects on expression induced by each stimulus—is a special case in gene control.^6^ Synergy has been described for multiple combinations of cytokines and extracellular signals including IFNγ/TNFα,^2^^,^^7^^,^^8^^,^^9^ interleukin-4/lipopolysaccharide,^10^ and IFNβ1/IFNγ.^11^ As a general property, synergy can occur in many cell types, though the specific gene expression programs induced may differ depending on cell lineage, developmental stage, tissue context, and cytokine combinations.^2^^,^^7^^,^^10^^,^^12^^,^^13^^,^^14^ At least two non-mutually exclusive models have been proposed to explain how synergy emerges: 1) TF cooperativity, in which DNA binding affinity of one TF is enhanced by local occupancy of another TF, leading to non-linear output of gene expression as a consequence of the combined TF action to recruit Pol II,^6^ or 2) kinetic transcription in which individual TFs stimulate distinct steps in the Pol II transcriptional cycle (i.e., initiation or elongation), which augments overall Pol II processivity.^15^^,^^16^

In addition to intrinsic transactivation functions, both STAT1- and p65-dependent transcription are potentiated via a dynamic interplay with chromatin-associated coactivators. The p300/CREB-binding protein (CBP) family of histone acetyltransferases physically interact with the transactivation domain (TAD) of STAT1 via their TAZ2 domains, increasing STAT1 acetylation in the TAD and thereby increasing DNA binding and transactivation activity.^17^^,^^18^ p300/CBP also associates with p65 via its Rel homology and C-terminal TADs.^19^^,^^20^ This interaction promotes reversible acetylation of p65 and histones locally, which increases transcriptional output.^21^ Specific acetylation of p65 on lysine residue 310 recruits BRD4, a member of the BET bromodomain-containing protein family via its bromodomain, an acetyl-lysine reader motif.^22^ Once recruited, BRD4 forms super enhancers (SEs) that drive dynamic increases in transcription of proinflammatory genes through recruitment of positive elongation factor-b (PTEF-b), which along with BRD4 phosphorylates Pol II on its C-terminal domain and releases Pol II from promoter-proximal pausing.^23^^,^^24^^,^^25^ Altering IFNγ- or TNFα-dependent transcriptional activation in certain types of cancers and SARS-CoV-2 through inhibition of p300/CBP and BET bromodomain proteins may also have clinical significance.^26^^,^^27^^,^^28^^,^^29^ Thus, chromatin-dependent signaling plays critical roles in communicating many of the transcriptional functions of STAT1 and p65 to Pol II. However, comparatively less is known about how chromatin coactivators contribute to synergism of gene expression and the associated cellular phenotypes induced by IFNγ and TNFα.

Vascular endothelium—the single-cell thick layer lining blood vessels—plays a critical role in cardiovascular health. In addition to homeostatic control of systemic blood pressure,^30^ thrombosis,^31^ and metabolism,^32^ endothelial cells (ECs) also serve as innate immune-like cells that transduce proinflammatory cytokine signals including IFNγ and TNFα, present antigen through major histocompatibility complexes, and regulate leukocyte trafficking to sites of injury or infection.^33^^,^^34^^,^^35^^,^^36^ As such, ECs control the tempo, magnitude, and propagation of inflammation locally and systemically. In this study we investigated transcriptional synergy and its dependencies during costimulation of human aortic ECs (HAECs) with IFNγ/TNFα. By integrating stable isotope quantification of nascent RNA, RNA sequencing (RNA-seq), and non-amplified mRNA analyses, we identified a small subset of proinflammatory genes including the chemokines *CXCL9*, *-10*, and *-11* that are synergistically induced by IFNγ/TNFα in 1 h. This synergism occurred even at non-saturating levels of cytokines. Prolonged IFNγ/TNFα costimulation increased chemokine protein levels and cell death. At a chromatin level, this resulted in formation of increased sites of accessibility in non-coding regions of synergistically induced genes and co-binding of STAT1 and p65. Cotreatment of HAECs with low-dose inhibitors targeting p300/CBP and BET bromodomain proteins synergistically abrogated chemokine gene induction and protein production. Thus, we demonstrate that the rapid induction of synergistic transcription by IFNγ/TNFα mediated by STAT1 and p65 is especially vulnerable to combinatorial disruption of chromatin-dependent signaling.

## Results

### IFNγ/TNFα costimulation of HAECs induces nascent RNA production and synergistic expression of proinflammatory chemokine genes

**Figure 1:**
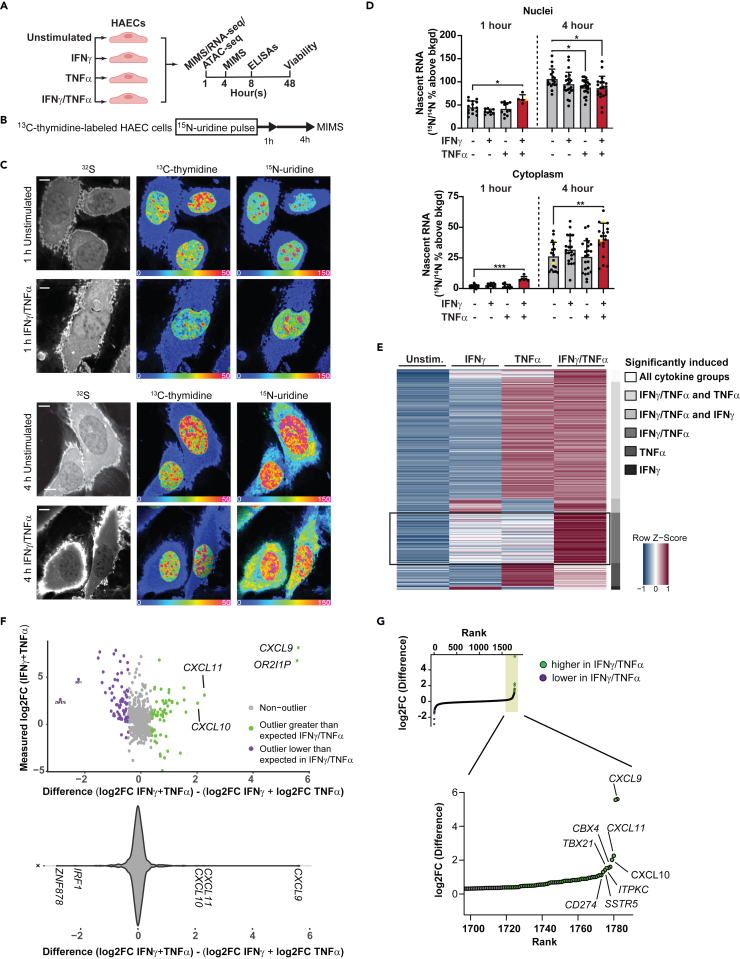
IFNγ/TNFα costimulation of HAECs induces nascent RNA production and synergistic expression of proinflammatory chemokine genes (A) Schematic depicting treatment groups and experimental time points in HAECs. (B) Schematic of experimental approach for MIMS experiments. (C) MIMS images of HAEC stimulated with IFNγ/TNFα for 1 (top) or 4 h (bottom) versus unstimulated cells. ^32^S images reveal cellular and nuclear contours (left column). Hue saturation intensity images are used to visually represent isotope ratio measurements and in turn map ^13^C-thymidine labeling of DNA (^13^C/^12^C, middle) and ^15^N-uridine labeling of nascent RNA (^15^N/^14^N, right). The lower bound of the color scale (blue) is set to natural background (^13^C = 1.1%; ^15^N = 0.37%) and the upper bound of the scale is set to reveal differential labeling expressed as % above natural background. Scale bars = 5 μm. See also Figure S1B. (D) Nuclear (top) and cytoplasmic (bottom) ^15^N-uridine labeling was quantified at the 1 and 4-h timepoints after stimulus and expressed as % above natural background. Each dot represents a different cell/nucleus with mean ± SD. One-way ANOVA, Dunnett’s test used for 4-h time point; Kruskal-Wallis/Dunn’s nonparametric test used for 1-h time point. ∗*p* < 0.05 ∗∗*p* < 0.01. Yellow dots correspond to the cells shown in the images in Figure 1C. (E) Heatmap of averaged differential gene expression in unstimulated HAECs and HAECs treated with IFNγ (50 ng/mL), TNFα (25 ng/mL) or IFNγ/TNFα (*n* = 4 per condition). (F) Scatterplot of difference in expression calculated by log_2_(FC) of IFNγ/TNFα (i.e., costimulated) minus the sum of the log_2_FC (IFNγ) plus log_2_FC (TNFα) (*n* = 4 per condition). Color indicates outlier status. All log2FC were calculated with cytokine vs. unstimulated cells. (G) Scatterplot of ranked log_2_(FC) difference between IFNγ/TNFα minus the sum of the FC observed in IFNγ plus FC observed in TNFα. Green color indicates gene expression greater in IFNγ/TNFα, blue color indicates gene expression is less in IFNγ/TNFα than the sum of the FC observed in IFNγ plus FC in TNFα (*n* = 4 per condition).

We first examined the functional consequences of combined IFNγ/TNFα signaling on global RNA regulation by utilizing multi-isotope imaging mass spectrometry (MIMS). MIMS leverages nanoscale secondary ion mass spectrometry to measure isotope ratios at suborganelle resolution Figure 1C). Incorporation of a stable isotope tracer is quantitatively captured by an increase in the isotopic ratio above the natural background ratio.^37^ We have previously used thymidine tracers to measure DNA synthesis and cell division.^38^^,^^39^ Here, we used ^13^C-thymidine to saturate DNA labeling in proliferating HAECs (label 14 days) and map intranuclear DNA architecture, followed by ^15^N-uridine pulse labeling concurrent with cytokine stimulation to capture changes in global nascent RNA synthesis (Figure 1B). Costimulation of HAECs with IFNγ/TNFα augmented ^15^N-uridine labeling that was detectable in the nucleus at 1 h and in the cytoplasm at 1 and 4 h. This effect was not observed in HAEC exposed to each individual cytokine stimulus (Figures 1D, S1A, and S1B). These data identify that the dynamic cellular response to INFγ/TNFα costimulation involves a rapid surge in global RNA synthesis, suggestive of a distinct regulatory interaction between IFNγ and TNFα at the level of gene expression.

Despite the quantitative and spatial power of MIMS to measure nascent RNA in cell compartments, it cannot resolve gene-level ^15^N-uridine signal needed to determine changes of individual transcripts in the nucleus. As such, we next determined the differential gene expression patterns in HAECs after 1 h stimulation with maximal concentrations of IFNγ, TNFα, or both using unbiased RNA-seq. We chose 1 h to focus on primary transcriptional effects and thereby avoid the secondary waves of gene regulation that arise during more prolonged cytokine stimulation. Differential gene expression analysis using pairwise comparisons of each cytokine vs. unstimulated cells identified the induction of 43 genes with IFNγ, 218 genes with TNFα, and 277 genes with IFNγ/TNFα, using a threshold of 2-fold change and adjusted *p* value <0.05 (Figures S1C–S1E). The synergy conditioned overlapped almost completely with TNFα stimulation at this time point. To investigate synergy-specific gene targets, we next examined the pattern of changes in expression using all genes upregulated by any cytokine by at least 2-fold. We row-normalized the data for each gene and compared across each treatment group (Figure 1E). This analysis revealed a group of 72 genes that were upregulated in the synergy treatment specifically, as compared to IFNγ or TNFα alone (Figure 1E). To determine whether the induction of these genes was different from the individual cytokine stimulations, we performed an outlier analysis. We compared the difference between the measured fold change in IFNγ/TNFα vs. unstimulated cells and the sum of the fold change for IFNγ vs. unstimulated plus TNFα vs. unstimulated cells (Figure 1F, top). This scatterplot revealed a small group of genes with very large differences, indicating fold changes for synergy conditions that far exceeded the predicted fold changes if the regulation was purely linear between the cytokine treatment groups. By graphing the gene density distribution, it was clear overall the fold changes followed a Gaussian (i.e., Normal) distribution with the vast majority of genes displaying no differences between cytokine groups and hence a difference score near zero (Figure 1F, bottom). This observation enabled us to identify genes whose fold changes clearly fell outside this normal distribution, thereby representing statistical outliers. Notably, we observed outliers in both positive (green) and negative (purple) directions, indicating expression of certain genes changed less than additively compared to each cytokine alone (purple dots), while the magnitude of change in expression of other genes was clearly much greater than predicted compared to individual cytokine results (green dots, *n* = 65). Formal ranking of these difference scores identified that the chemokines *CXCL9*, *-10*, and *-11* were among the most extreme outliers in terms of positive gene induction/fold change (Figure 1G). Based on these outlier analyses, we concluded that these genes represented a distinct group that was induced synergistically by dual cytokines.

### Transcriptional synergy elicited by IFNγ/TNFα signaling requires STAT1 and p65

**Figure 2:**
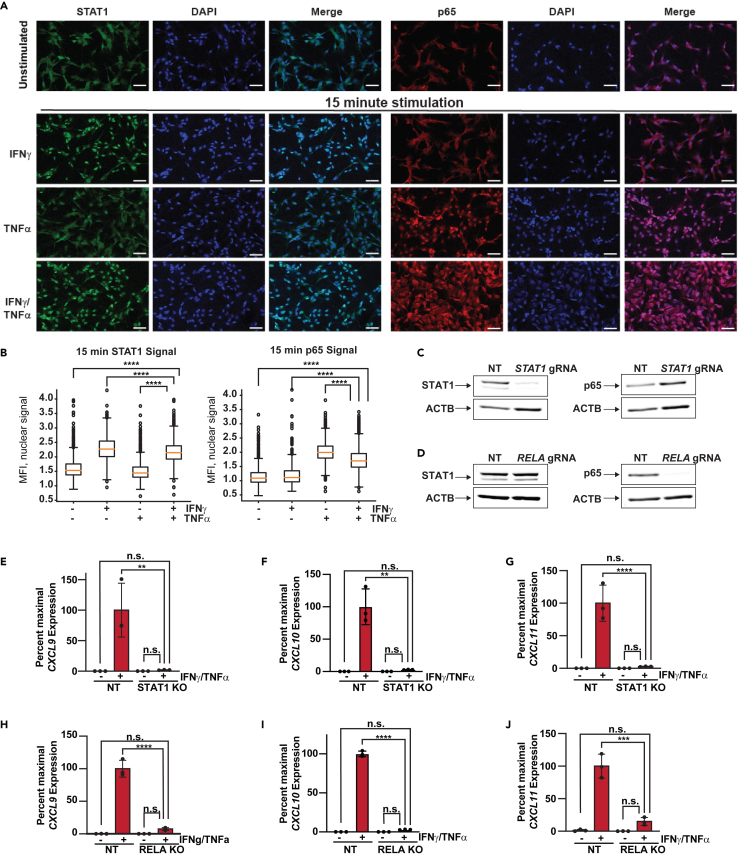
Transcriptional synergy elicited by IFNγ/TNFα signaling requires STAT1 and p65 (A) Representative photomicrographs of STAT1 (green) and p65 (red) immunofluorescence in TeloHAECs treated with vehicle or combined IFNγ/TNFα for 15 min. Nuclei are indicated by DAPI staining (blue color). All samples were imaged at 10X magnification on Zeiss confocal microscope. Scale bar = 100 μm. (B) Boxplots for quantification of images in (A). Kruskal-Wallis test was performed followed by a pairwise Wilcoxon test to determine statistical differences between treatment groups. Box indicates first quartile (top), median (middle, orange), and third quartile (bottom), whiskers indicate minium and maxium values in the range. ∗∗∗*p* value <0.001; ∗∗∗∗*p*-value <0.0001. (C and D) Western blots of STAT1 and p65 in *STAT1* KO (C) or *RELA* KO (D). ACTB is a loading control. (E–G) Bar graphs of *CXCL9* (E), *CXCL10* (F), and *CXCL11* (G) expression in *STAT1* KO HAECs unstimulated or treated with IFNγ/TNFα. Data shown are mean ± SD (*n* = 3 per condition). ∗∗*p* values <0.01; *p* value <0,0001. (H–J) Bar graphs of *CXCL9* (G), *CXCL10* (H), and *CXCL11* (I) expression in *RELA* KO HAECs unstimulated or treated with IFNγ/TNFα. Data shown are mean ± SD (*n* = 3 per condition). ∗∗∗*p* value < 0.001; ∗∗∗∗*p* value <0,0001.

IFNγ and TNFα signal transduction pathways are known to activate STAT1 and p65/NF-κB, respectively. Crosstalk between IFNγ and TNFα has also been reported between these two pathways.^40^ Thus, we first examined the impact of individual cytokine stimulation on STAT1 or p65 nuclear translocation—a key step in the activation of these TFs. Subcellular localization of each TF was tracked over time in response to cytokine stimulation using immunofluorescence. IFNγ or TNFα each stimulated movement of STAT1 or p65, respectively, into the nucleus within 15 min (Figures 2A and 2B). This shift persisted over 1 and 4 h (Figures S2A and S2B). Costimulation with IFNγ/TNFα had similar effects on STAT1 and p65 translocation as compared to individual cytokines.

To further investigate the specific roles of STAT1 and p65 in regulating synergy, we used CRISPR-Cas9 to generate stable knockout (KO) lines of each TF in HAECs. Electroporation of cells with Cas9-Ribonucleoprotein complexes containing single guide RNAs (sgRNAs) targeting each TF achieved 92% and 94% KO of STAT1 and p65, respectively, at the protein level (Figures 2C and 2D). A non-target (NT) sgRNA was included as a negative control for comparison. Given the near-complete KO effect, we used these polyclonal lines to assess TF dependency in cytokine-mediated gene regulation. STAT1 KO resulted in a 98%, 97%, and 97% decrease in the maximal induction of *CXCL9*, *-10*, and *-11* (Figures 2E–2G, the fold change values are provided in Table S1), respectively, while p65 KO reduced maximal activation of *CXCL9*, *-10*, and *-11* by 92%, 97%, and 85%, respectively (Figures 2H–2J; the fold change values provided in Table S1). It is possible that cytokines can alter mRNA stability.^41^ To address this point, we performed cytokine washout experiments. ECs were treated with dual cytokines for 1 h and then switched to media without cytokine for 3 h. RNA was harvested at 4 h for all conditions. By qPCR, transcripts were decreased by 50%–80%, as compared to 4-h synergy condition, suggesting that stabilization of RNA could not explain all observed effects (Figures S2F–S2I). Notably, several other TFs including interferon regulatory factors (IRFs) are induced by IFNγ and TNFα (Figure 1E) albeit sub-additively. However, the observed synergistic response did not require new protein synthesis, as determined by cycloheximide cotreatment during cytokine stimulation, suggesting that newly induced TF protein expression was not critical for gene induction (Figure S2J). Collectively, these data demonstrate that both STAT1 and p65 are required for the transcriptional synergy response by IFNγ/TNFα.

### Synergistic induction of proinflammatory genes is not dependent on saturation of IFNγ or TNFα signaling

**Figure 3:**
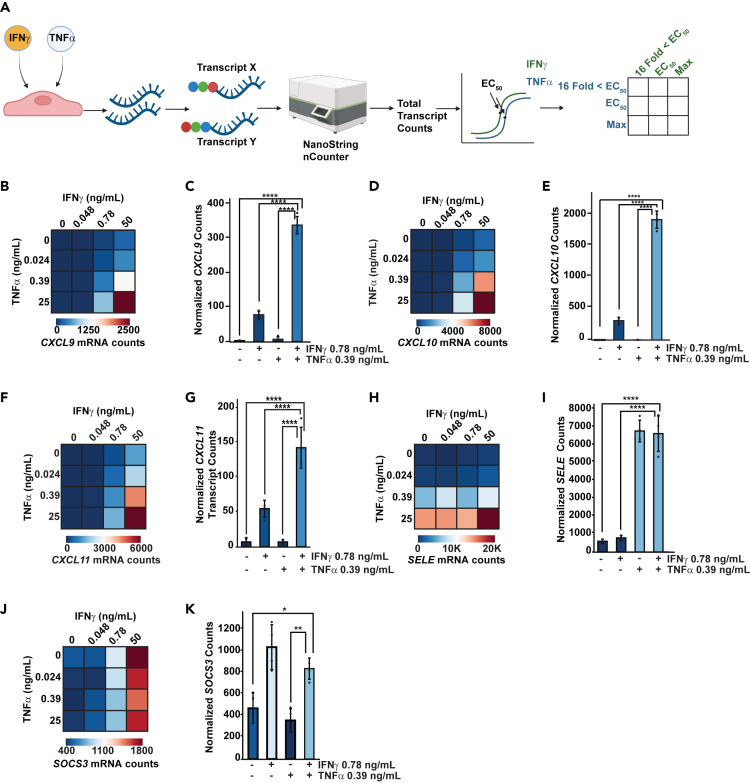
Synergistic induction of proinflammatory genes is not dependent on saturation of IFNγ or TNFα signaling (A) Schematic of NanoString workflow, identification of EC_50_ for IFNγ and TNFα (Figure S3A), and experimental design of dual-cytokine dose-response experiments. (B, D, F, H, and J) Heatmap of *CXCL9* (B), *CXCL10* (D), *CXCL11* (F), *SELE* (H), and *SOCS3* (J) expression in response to maximal concentrations, EC_50_ and 16-Fold < EC_50_ of cytokine responsive genes of IFNγ and/or TNFα (*n* = 4 per condition). (C, E, G, I, and K) Bar plot of normalized *CXCL9* (C), *CXCL10* (E), *CXCL11* (G), *SELE* (I), and *SOCS3* (K) transcript counts in Figures 2B–2D, 2F, 2H, and 2J. Bars indicate mean ± SD (*n* = 4 per condition). One-way ANOVAs were performed followed by Tukey’s *post hoc* analysis to determine statistical differences between treatment groups. ∗*p* < 0.05, ∗∗*p* < 0.01, ∗∗∗∗*p* ≤ 0.0001.

The experiments described utilized saturating concentrations of IFNγ and TNFα, which raises a key question: does synergy occur simply as a function of maximal activation of p65 and STAT1? Furthermore, RNA-seq results are semi-quantitative and fold change measurements can be misleadingly large when baseline levels of gene expression are low. To circumvent these issues, we leveraged the NanoString platform that directly counts mRNA transcripts quantitatively without RNA amplification, thereby avoiding potential analytical challenges arising with relative expression changes determined by RNA-seq (Figure 3A). We designed a custom NanoString probe set that included 3 housekeepers (*GUSB*, *HPRT1*, and *NOL7*), five genes that are putatively synergistic (*CXCL9*, *CXCL10, CXCL11*, *CX3CL1*, and *IL32*) and two genes that are responsive to IFNγ or TNFα only (*SOCS3* and *VCAM1 or SELE*, respectively), based on the RNA-seq data. The gene expression changes elicited by each cytokine were first investigated by generating a 12-point dose-response curve for IFNγ or TNFα treatment in HAECs. (Figure S3A). From these data the half-maximal effective concentration (EC_50_) for each cytokine was extrapolated: EC_50_ for IFNγ = 7.8 ng/mL and EC_50_ for TNFα = 0.39 ng/mL. Based on these analyses, HAECs were then treated with IFNγ and/or TNFα at maximal, EC_50_, or concentrations 16-fold lower than EC_50_, and transcript counts for genes were measured in our probe set. Synergistic induction of *CXCL9*, *-10*, and *-11* was evident at concentrations as low as 0.78 ng/mL of IFNγ and 0.39 ng/mL of TNFα (Figures 3B–3G). Similar patterns of synergy were observed with all concentrations of TNFα when combined with maximal IFNγ (Figures S3B–S3J). We observed no synergistic induction of *SELE* or *SOCS3* at any combination of IFNγ and TNFα (Figures 3H–3K). These results indicate that STAT1 cooperates with p65 to synergistically induce genes at sub-saturating levels of cytokine activation.

### Prolonged IFNγ/TNFα costimulation functionally synergizes to increase chemokine protein and cell death in HAECs

**Figure 4:**
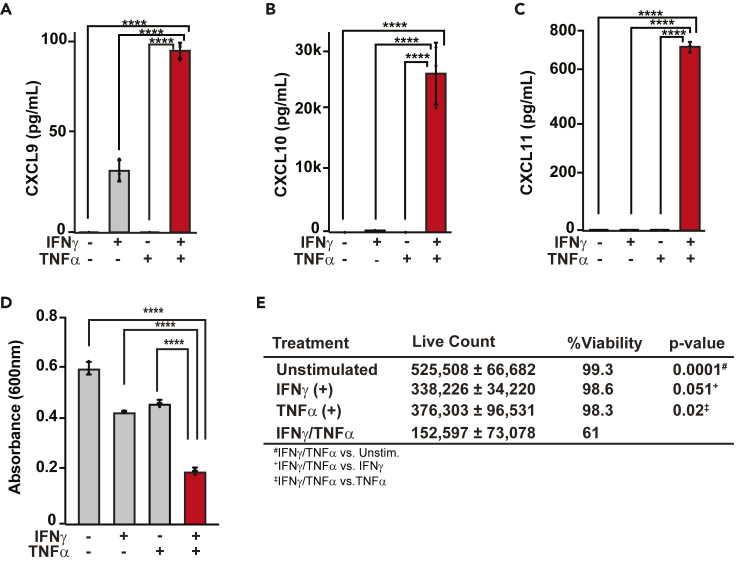
Prolonged IFNγ/TNFα costimulation functionally synergizes to increase chemokine protein and cell death in HAECs (A–C) Bar plots of secreted protein for CXCL9 (A) CXCL10 (B) and CXCL11 (C). Data shown are mean ± SD (*n* = 3 per condition). (D) Bar plot of absorbance at 600nm as an indicator of cell viability as measured by an MTT assay in unstimulated HAECs and HAECs treated with IFNγ, TNFα, or IFNγ/TNFα. Data shown are mean ± SD (*n* = 3 per condition). (E) Table containing means of viable cell counts ± SD in HAECs treated with vehicle or cytokines for 48 h (*n* = 3). One-way ANOVA followed by a Tukey’s post hoc analysis was utilized to determine statistical significance for cell count. *p-*value comparisons are listed underneath the table. For (A–D) one-way ANOVAs were performed followed by a Tukey’s *post hoc* analysis to determine statistical differences between treatment groups. ∗∗∗∗*p* ≤ 0.0001.

The dynamic changes in chemokine transcripts induced by INFγ/TNFα motivated us to examine whether protein levels also change. As these chemokines serve critical biological roles in immune cell activation when released from cells, we performed enzyme-linked immunosorbent assays (ELISAs) to quantify the secreted proteins in the supernatant of HAECs.^42^ Baseline levels of CXCL9, -10 or -11 were below the limit of detection in the ELISA (lower limit: CXCL9 = 31.2 pg/mL, CXCL10 = 7.8 pg/mL, CXCL11 = 62.5 pg/mL). IFNγ stimulation for 8 h increased CXCL9 to 35 pg/mL and CXCL10 to 315.99 pg/mL, while CXC11 protein levels remained less than assay (Figures 4A–4C). No induction for any chemokine was detected in response to TNFα, which is consistent with the absence of an effect on gene expression (Figures 4A–4C). Following IFNγ/TNFα costimulation, multiplicative increases in the protein levels of CXCL9 (97.3 pg/mL), CXCL10 (27,097 pg/mL), and CXCL11 (711 pg/mL) occurred relative to the levels following either cytokine alone (Figures 4A–4C). These results align with transcript data in which *CXCL10* had the highest transcript counts on NanoString and the highest reads per kilobase million (RPKMs) in RNA-seq, followed by *CXCL11* and then *CXCL9* (Figures 1F, 3B–3G, and S3B–S3J).

Previous work has demonstrated that prolonged (>24 h) IFNγ/TNFα signaling results in death of multiple cell types including ECs via PANoptosis.^2^^,^^43^^,^^44^ To test this idea in our model, we compared cell counts and viability of unstimulated HAECs versus cells stimulated with IFNγ, TNFα, or IFNγ/TNFα cytokines for 48 h. In unstimulated HAECs, counts doubled over the course of the experiment, consistent with normal growth and doubling times of these cells. Single cytokine-treated cells also proliferated, but to a lesser degree. By contrast, IFNγ/TNFα stimulation decreased total counts compared to starting values, culminating in 39% cell death relative to other groups cells (Figure 4E). The combination of decreased overall count and viability in the IFNγ/TNFα-stimulated cells reveals that prolonged dual cytokine stimulation is toxic in HAECs (Figure 4E). Overall, these data demonstrate that more prolonged IFNγ/TNFα stimulation drives large increases in these chemokine proteins that are known to play key roles in systemic inflammatory responses, and the overall activation ultimately culminates in significant cell death.

### Dual cytokine stimulation results in the formation of accessible elements and recruitment of STAT1 and p65 at the *CXCL9*, *-10*, and *-11* locus

**Figure 5:**
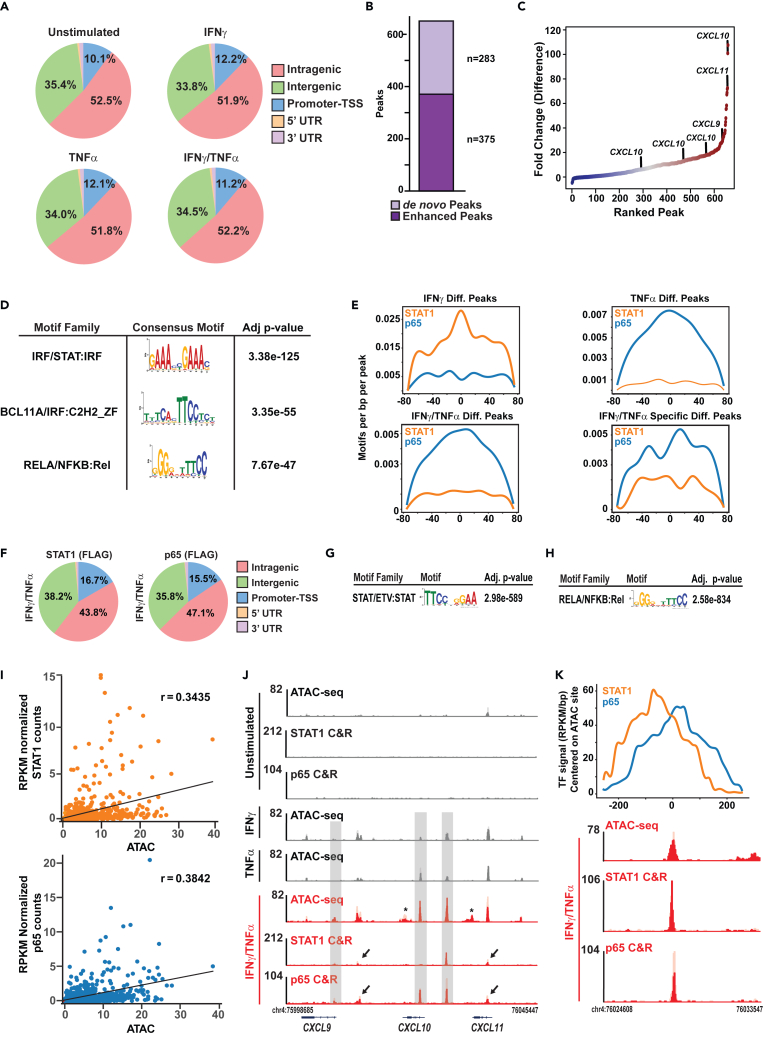
Dual-cytokine stimulation results in the formation of *de novo* accessible elements at the *CXCL9*, *-10*, and *-11* locus and recruitment of STAT1 and p65 (A) Pie chart of genomic localization of ATAC-seq peaks as a percentage of the total peaks. Unstimulated (upper left), IFNγ (upper right), TNFα (lower left), and IFNγ/TNFα (lower right) (*n* = 2 per condition). (B) Stacked bar plot depicting number of enhanced (dark purple) or *de novo* (light purple) accessible regions in peaks differential to IFNγ/TNFα. (C) Rank plot of log_2_(RPKM) ATAC-seq signal in SSAEs. (D) Position weight matrix plots of the top motifs found in the IFNγ/TNFα-specific differential peaks. (E) Signal plots of p65 or STAT1 motif density distribution in differential peaks in each cytokine condition. (F) Pie charts of genomic localization of CUT&RUN peaks for FLAG-STAT1 and FLAG-p65. (FLAG) indicate the antibody used for CUT&RUN. (G and H) Position weight matrix plots of the top motif found in the STAT1 peaks (G) and p65 peaks (H) in IFNγ/TNFα stimulated HAECs. (I) Scatterplots of STAT1 signal (top, orange) and p65 signal (bottom, blue) vs. ATAC signal at SSAEs. R value indicates Pearson correlation coefficient. (J) Gene tracks of ATAC-seq and CUT&RUN (C&R) for STAT1 and p65 at the *CXCL9*, *-10*, and *-11* locus in HAECs at baseline and in response to IFNγ, TNFα or both (red). *N* = 2 per condition. Replicates are shown in two shades of color on the same track. Y axis is rpm/bp. Gray boxes indicate SSAEs co-bound by STAT1 and/or p65; asterisks indicate SSAEs with no TF co-binding; arrows indicate STAT1 and/or p65 binding at ATAC-seq sites that are not categorized as SSAEs. (K) Gene track zoomed in on the *CXCL10* and *-11* intergenic enhancer showing the close proximity of STAT1 and p65 co-binding at this location centered on the ATAC-seq SSAE.

After determining changes in gene, protein, and cell phenotypes in response to IFNγ/TNFα costimulation, we next investigated the chromatin landscapes in HAECs. ATAC-seq was performed in HAECs stimulated with or without IFNγ, TNFα, or IFNγ/TNFα for 1 h (*n* = 2 for each condition), and reads were filtered on nucleosomal-free regions (<150 bp). All four conditions had similar numbers of peaks (unstimulated = 186,612 peaks, IFNγ = 154,313 peaks, TNFα = 154,252 peaks, and IFNγ/TNFα = 168,185 peaks). The overall distribution of ATAC-seq peaks at promoter-transcriptional start sites (TSS), 5′-untranslated region (UTR), 3′-UTR, intragenic, or intergenic regions did not differ between groups (Figures 5A and S4A–S4D).

We next investigated dynamic changes in chromatin accessibility in response to cytokines. When compared to unstimulated cells, statistically significant increases in regions of open chromatin were evident in all cytokine groups: IFNγ (5,105 peaks), TNFα (9,778 peaks), and IFNγ/TNFα (13,738 peaks) using a fold change threshold of 1.5 and *p*-value <0.0001 (Figures S4E–S4G). The total number of differential peaks suggested considerable overlap in accessible elements. Our goal was to determine whether any unique chromatin features could be identified in HAECs treated with IFNγ/TNFα. Thus, we next performed 3 pairwise analyses: IFNγ/TNFα versus 1) unstimulated, 2) IFNγ-stimulated, or 3) TNFα-stimulated HAECs. 658 peaks were significantly enriched in synergy conditions compared to IFNγ or TNFα, of which 375 were enhanced (i.e., increased compared to individual cytokine) and 283 were *de novo* sites (i.e., not present in single cytokine) (Figures 5B and S4E–S4G). We designated these 658 regions as synergy-specific accessible elements (SSAEs). Ranking of SSAEs by signal identified several sites in the *CXCL9*, *-10*, and *-11* locus that were among the top peaks in the IFNγ/TNFα group (Figure 5C). Motif enrichment analysis confirmed that STAT1 and p65 were among the most enriched TF motifs in these regions (Figure 5D).

TF cooperativity is one mechanism posited to explain transcriptional synergy.^6^ To evaluate this in ECs, we generated histograms of p65 and STAT1 motif density centered on ATAC-seq summits. Both STAT1 and p65 motifs were enriched in the differential ATAC sites detected in IFNγ and TNFα groups, respectively. The p65 profile was more pronounced than STAT1 in the IFNγ/TNFα group (Figure 5E), consistent with the RNA-seq results, which showed TNFα drove a large proportion of the changes in gene expression with synergy. There was also a strong alignment between the summit of the peak and the apex of STAT1 and/or p65 motif (Figure 5E). The pattern of p65 and STAT1 motifs was distinctive in the IFNγ/TNFα differential peaks, in which they were spaced apart by ∼20 bp periodicity. Both IFNγ and TNFα can direct the formation of SEs—exceptionally large regions of *cis*-regulatory DNA that are densely bound by TFs.^23^^,^^45^ However, in this dataset the overall ATAC signal, peak length, and signal density at distal elements and gene bodies were not different between all cytokine-stimulated groups (Figures S4H and S4I); and most regions had similar chromatin accessibility in single- and dual-cytokine conditions (Figure S5A). The overall correlation between SSAEs and RNA-seq was weak, but there was a statistically significant increase in ATAC-seq signal at genes that were deemed outliers from our computational analysis (Figure S5B) and 46% of outlier genes (30 of 65) had an SSAE within 500 kb of the transcriptional start site (Figure S5C).

Based on the chromatin accessibility predictions, we next directly mapped the DNA binding of STAT1 and p65 in response to IFNγ/TNFα. We first attempted “cleavage under targets and release under nuclease” (CUT&RUN) using antibodies for the endogenously expressed proteins, but the results were inconsistent. To overcome this technical problem, we engineered stable HAEC lines to express either N-terminal 3xFLAG-STAT1 or 3xFLAG-p65 via lentivirus in STAT1 or p65 single KO cells, respectively, which we had already validated in Figure 2B. The protein expression levels of the FLAG-tagged STAT1 and p65 were similar to wild-type cells (i.e., STAT1+/p65+ cells) and were equally responsive to dual-cytokine stimulation (Figures S5D and S5E). Anti-FLAG CUT&RUN identified clear recruitment and binding of STAT1 (3056 sites) and p65 (4355 sites) in response to IFNγ/TNFα as compared to unstimulated cells. Genomic localization of the TFs was distributed among intergenic, intragenic, and TSS regions (Figure 5F), and the top motif in 3xFLAG-STAT1 cells or 3xFLAG-p65 cells was STAT1 (Figure 5G) or p65 (Figure 5H), respectively. Co-binding analysis showed that 17% of *de novo* ATAC-seq sites were co-bound by one or both TFs; this percentage increased to 31% when using only the enhanced SSAEs. Overall, there was a moderate correlation between TF signal and SSAEs (Figure 5I). At the *CXCL9*, *-10*, and *-11* locus there was clear evidence of co-binding at 3 SSAEs (Figure 5J, gray boxe**s**). Two other sites were co-bound by STAT1 and p65, with clear ATAC-seq signal, which were not classified as SSAEs (Figure 5J, arrows). Notably, close spacing of STAT1 and p65 binding at some of these co-bound regions was consistent with the ATAC motif predictions (Figure 5K). To examine the interdependency of STAT1 and p65 recruitment to this region, we next performed CUT&RUN in cells with KO of both TFs that were then reconstituted with either 3xFLAG-STAT1 or 3xFLAG-p65. As before, protein expression was similar to wild-type levels (Figure S5F). Following dual-cytokine stimulation, overall binding of p65 and STAT1 was predominantly unaffected genome-wide with only 48 STAT1 sites (1.6%) and 293 p65 sites (6.7%) decreased after KO of the opposite TF (Figure S5G). Most regions including at canonical IFNγ (*SOCS3*) and TNFα (*CCL2*) enhancers were not altered (Figures S5H and S5I). At the *CXCL9*, *-10*, and *-11* locus, STAT1 recruitment did not change with p65 KO; however, p65 recruitment at the *CXCL9* and -*10* promoters and intergenic sites between *CXCL9* and *-10* and *CXCL10* and *-11* was completely lost in cells lacking endogenous STAT1 (Figure S5J arrows). These data demonstrate that STAT1 is a critical determinant of p65 recruitment to select genes in IFNγ/TNFα-induced transcriptional synergy.

### Synergistically induced genes harbor transcriptional dependencies on p300/CBP and BET-bromodomain-containing proteins

**Figure 6:**
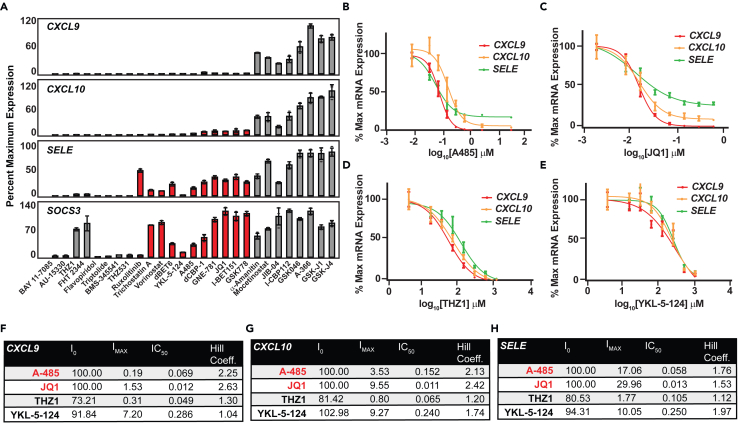
Synergistically induced genes harbor transcriptional dependencies on p300/CBP and BET bromodomain-containing proteins (A) Bar plots of gene expression of *CXCL9* (top), *CXCL10* (2nd from top), *SELE* (2nd from bottom), and *SOCS3* (bottom) comparing untreated cells versus cells costimulated with IFNγ/TNFα+Vehicle or maximal concentrations (10μM) of transcriptional inhibitors. For AU-15330 and FHT1 samples were pretreated at 1 μM for 1 h before adding cytokine. Data shown are mean ± SD (*n* = 3 per condition). (B–E) Dose-response curves for A-485 (B), JQ1 (C), THZ1 (D), and YKL-5-124 (E) in HAECs stimulated with IFNγ/TNFα and inhibitors (1 h). Graphs show % maximal expression (y axis) for *CXCL*9 (red line), *CXCL10* (yellow line) and SELE (green line) vs. log_10_ inhibitor dose (x axis). Data are reported as mean ± SD (*n* = 4 per observation). (F–H) Tables of biochemical parameters calculated from dose-response curves for each gene from (B–E).

Changes in accessibility implicate chromatin regulators in these synergy transcriptional responses. Indeed, the STRING database of protein-protein interactions for STAT1 and p65 confirms interactions between coactivators including BRD4—a member of the BET bromodomain-containing protein family—and p300/CBP as well as multiple histone modifiers (deacetylases, acetyltransferases, and methyltransferases) (Figures S6A and S6B).To explore coactivator dependencies more systematically, we developed a targeted chemogenomic miniscreen using 27 small-molecules inhibitors targeting coactivators, histone-modifying enzymes, nucleosome-remodeling proteins, components of the general transcriptional machinery, and key nodes in the IFNγ or TNFα signaling pathways. HAECs were stimulated with IFNγ/TNFα in the presence or absence of each compound for 1 h. Following RNA extraction, *CXCL9*, *-10*, *SELE*, and *SOCS3* mRNA levels were measured by real-time qPCR. As expected, small-molecule inhibitors of IFNγ-JAK-STAT (Ruxolitinib) or TNFα signaling (BAY) completely abrogated *SOCS3* and *SELE* expression, revealing fidelity of the gene responses to each cytokine pathway (Figure 6A). The screen further identified ten small-molecule inhibitors that more selectively reduced induction of *CXCL9* and *CXCL10* compared to IFNγ- or TNFα-responsive genes (*SOCS3* or *SELE*, respectively) (Figure 6A, red). Of these more selective inhibitors, four directly targeted the BET bromodomain protein family (BRD2, 3 ,4) and three targeted the p300/CBP family (Figure 6A). Surprisingly, targeted inhibition of BET bromodomain 1 (BD1 = GSK778) but not bromodomain 2 (BD2 = GSK046) selectively inhibited *CXCL9* and *-10* (Figure 6A), while BD2 had much less of an effect overall. This result differs from prior reports that implicate BD2 specifically in signal-responsive transcription.^46^ Inhibition of p300/CBP lysine acetyltransferase (KAT) activity (A-485) or the p300/CBP bromodomain (GNE-781) as well as targeted protein degradation of p300/CBP (dCBP-1) all resulted in more selective inhibition of *CXCL9* and *-10* induction (Figure 6A).

Guided by the known STAT1 and p65 protein-interaction profiles (Figures S6A and S6B), we focused on the inhibitors for p300/CBP lysine acetyltransferase function (A-485) and the BET-family of bromodomain-containing proteins (JQ1, pan-BET bromodomain inhibitor) to perform more in-depth biochemical modeling. Eight-point dose-response curves were generated for A-485 and JQ1 and determination of the baseline inhibition (I_0_), IC_50_, maximal inhibition (I_max_), and Hill coefficients (HCs) of each gene in response to each inhibitor (Figures 6B and 6C–6H). Since both p300/CBP and BRD4 are known to play essential roles in transcription more broadly, we wanted to ensure that the effects were not resulting simply from generalized inhibition of all transcription. As such we included additional dose-response curves for THZ1 (CDK-7, -12, and -13 inhibitor) and YKL-5-124 (CDK7 inhibitor) for comparisons and to assist in the biochemical modeling of unique transcriptional dependencies for *CXCL9* and *CXCL10*—two of the exemplary synergy genes (Figures 6D and 6E). While there were no differences in the A-485 and JQ1 IC_50_ data when comparing synergistically induced genes with *SELE* (*CXCL9* 68.5 nM; 12 nM, *CXCL10* 152 nM; 11 nM, *SELE* 57.6 nM; 13 nM), there were differences in the I_max_ values with *CXCL9* (0.19%, 1.53%) and *CXCL10* (3.53%, 9.55%), nearly completely inhibited at high concentrations of A-485 and JQ1, respectively (Figures 6F and 6G). By comparison, I_max_ values for *SELE* (17.06%, 29.96%) revealed decreased efficacy of JQ1 (Figure 6H). In contrast to these results, THZ1 and YKL-5-124 strongly inhibited all genes measured based on I_max_ values: THZ1:(*CXCL9* 0.31%, *CXCL10* 0.80%, *SELE* 1.77%) and YKL-5-124: (*CXCL9* 7.20%, *CXCL10* 9.27%, *SELE-*10.05%) (Figures 6D–6H). These data suggest that the observed effects of A-485 and JQ1 may not be caused by global effects on transcription.

The I_max_ data identified that p300/CBP KAT and BET bromodomain inhibition possessed high efficacy to abrogate *CXCL9* and *-10* induction. To determine the sensitivity of gene induction to these inhibitors, we determined the HCs—indicative of the extent of cooperativity of a drug or inhibitor to alter a stimulus-coupled response. HCs were calculated by generating a 4-parameter logistic fit on dose-response data for *CXCL9*, *CXCL10*, and *SELE* induction by IFNγ/TNFα during A-485 or JQ1 cotreatment. These calculations revealed that *CXCL9* and *CXCL10* display ultrasensitivity to A-485, indicated by higher HC values (HC = 2.25 (*CXCL9*), = 2.13 (*CXCL10*), = 1.76 (*SELE*)) and JQ1: HC = 2.63 (*CXCL9*), = 2.42 (*CXCL10*), = 1.53 (*SELE*) (Figures 6F–6H)).

We next assessed how treatment of HAECs with A-485 or JQ1 functionally impacts the secretion of CXCL9, -10 and -11 protein. HAECs were cotreated with IFNγ/TNFα and maximal concentrations of A-485 (2,000 nM) or JQ1 (500 nM) for 8 h. A-485 completely abrogated CXCL9 and CXCL11 induction and inhibited CXCL10 protein levels by 79.19% (Figures S6C–S6E). CXCL9 induction was completely inhibited after cotreatment with JQ1 while CXCL10 and CXCL11 displayed a 79.64% and 85.91% reduction, respectively, in protein levels in the presence of JQ1. These effects on protein suggested that A-485 or JQ1 might ameliorate other effects of IFNγ/TNFα synergy. To test this idea, cells were costimulated with IFNγ/TNFα and varying concentrations of A-485 or JQ1 for 48 h, after which the MTT (3-[4,5-dimethylthiazol-2-yl]-2,5 diphenyl tetrazolium bromide) assay was performed to assess cell death. We observed a significant decrease in viability after 48 h of IFNγ/TNFα treatment, which is in line with our previous data and published literature (Figure 4E).^2^ A-485 and JQ1 both partially rescued cell viability in a concentration-dependent manner. The lowest concentration for a detectable effect was 125 nM for A-458 and 62.5 nM for JQ1 (Figure S6F). Collectively, these data provide evidence that A-485 or JQ1 partially inhibited the toxic effects of IFNγ/TNFα dual stimulation in HAECs.

### p300/CBP or BET bromodomain inhibition does not alter chromatin accessibility or p65 and STAT1 recruitment to chromatin

**Figure 7:**
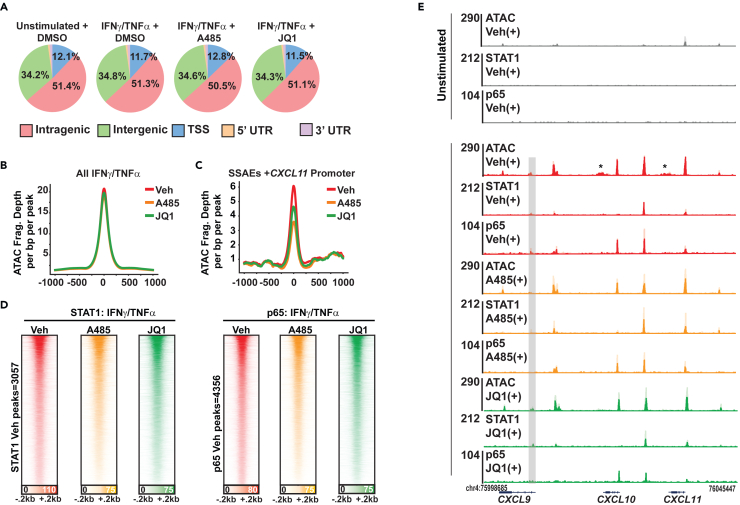
p300/CBP or BET bromodomain inhibition does not alter chromatin accessibility or p65 and STAT1 recruitment to chromatin (A) Pie charts of genomic localization of ATAC-seq peaks cotreated with IFNγ/TNFα with A-485 or JQ1. (B) Signal alignment plots of ATAC-seq costimulated with IFNγ/TNFα along with Vehicle, A-485 (2 μM) or JQ1 (500 nM), binned on all peaks present in IFNγ/TNFα + Vehicle. (C) Signal alignment plots of ATAC-seq cotreated with IFNγ/TNFα and Vehicle, A-485 (2 μM) or JQ1 (500 nM), binned on SSAEs only. (D) Heatmap alignments of STAT1 (left side) or p65 (right side) signal in Vehicle (red), A-485 (orange) or JQ1-treated (green) samples. All samples were stimulated with IFNγ/TNFα. All samples were aligned to the Synergy + Vehicle condition. (E) Gene tracks of *CXCL9*, *10*, and *11* locus showing ATAC-seq, p65 CUT&RUN (C&R) and STAT1 CUT&RUN in synergy conditions cotreated with Vehicle (Red), A-485 (orange) or JQ1 (green). Tracks for unstimulated cells are shown at the top in black. *N* = 2 replicates. Replicates are shown in two shades of color on the same track. Gray box indicates ATAC-seq site lost with A-485. Asterisks indicate ATAC SSAEs without TF binding that are lost with A-485 or JQ1 treatment.

Due to the strong inhibitory effects of A-485 and JQ1 on synergy genes, we investigated how they affect chromatin accessibility and genomic recruitment of STAT1 and p65, respectively. As compared to vehicle-treated cells, the distribution of ATAC-seq signal in inhibitor-treated HAECS was similar genome-wide (Figure 7A). Using all ATAC-seq sites in vehicle-treated samples, the signal alignment in the inhibitor-treated cells overlapped almost identically compared to vehicle (Figure 7B). When focusing only on SSAEs, there was a small decrease in signal in the A-485-treated group compared to JQ1 or vehicle, suggesting a modest effect of p300/CBP inhibition at SSAE sites, though not complete loss of accessibility (Figure 7C).

To assess the effect of p300/CBP or BET bromodomain inhibition on genomic localization of each TF, we performed CUT&RUN using 3XFLAG-STAT1 or 3XFLAG-p65 cells cotreated with IFNγ/TNFα with or without A-485 or JQ1. Signal alignments were superimposed when comparing the groups aligned on IFNγ/TNFα vehicle (Figure 7D) as well as genomic distribution of peaks (Figures S7A and S7B). Differential peak analysis via HOMER did reveal that 23 out of 3,057 peaks (0.75%) were decreased with A-485 and 9 out of 3,057 (0.29%) decreased with JQ1 for STAT1; for p65 a decrease in 52 out of 4,356 peaks (1.22%) with A-485 and 234 out of 4,256 peaks (5.37%) with JQ1 were detected. All together, these results suggest that global recruitment of p65 and STAT1 was unaffected by the presence of A-485 or JQ1, consistent with prior reports that these small-molecules target coactivators without affecting TFs. Examination of the *CXCL9*, *-10*, and *-11* locus specifically demonstrated that both p65 and STAT1 were recruited in response to dual cytokines and overlapped multiple ATAC-seq sites including SSAEs (Figure 7E) except for p65 binding at the *CXCL9* promoter (Figure 7E, gray boxes). The remainder of p65 and STAT1 occupancy profiles were insensitive to either A-485 or JQ1 (Figure 7E). Similar results were observed at *SELE* and *SOCS3* (Figures S7C and S7D). With respect to chromatin accessibility, two SSAEs that were not co-bound by STAT1 or p65 were decreased with A-485, and one with JQ1. These sites were located at the 3′ UTRs of *CXCL10* and *CXCL11*, perhaps indicative of the overall transcriptional effects of A-485 and JQ1 (Figure 7E, asterisks). These data indicate that the mechanism of transcriptional inhibition by A-485 and JQ1 does not involve complete displacement of STAT1 or p65 from *cis*-regulatory DNA at these regions.

### Synergy genes are sensitive to combined p300/CBP and BET bromodomain inhibition

**Figure 8:**
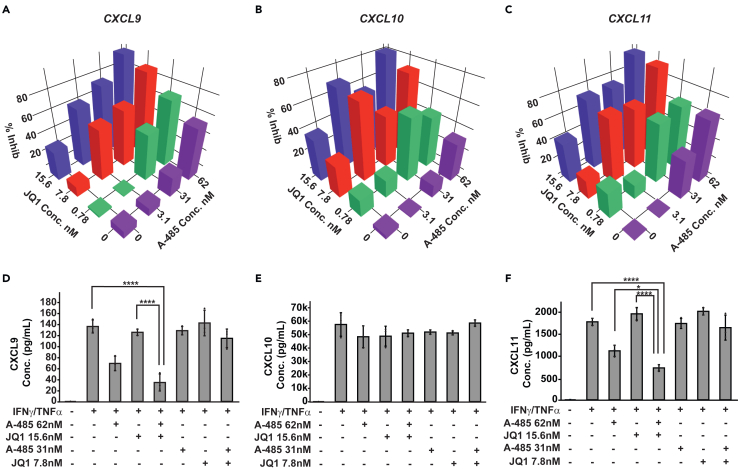
Synergy genes are sensitive to combined p300/CBP and BET Bromodomain inhibition (A–C) 3D bar plots of normalized transcript counts for *CXCL9* (A), *CXCL10* (B), and *CXCL11* (C) with the indicated amounts of A-485 and/or JQ1 (*n* = 3 per condition). (D–F) Bar plots listing supernatant concentrations of CXCL9 (D) CXCL10 (E) and CXCL11 (F) in IFNγ/TNFα + Vehicle-treated cells or with costimulation across various concentrations of A-485 and/or JQ1. A one-way ANOVA followed by Dunnett’s test was performed to assess statistical significance. Data shown are mean ± SD (*n* = 3 per condition). ∗*p* < 0.05 ∗∗∗∗*p* ≤ 0.0001.

Considering the differences in HCs for A-485 and JQ1 and their distinct mechanisms of action, we next hypothesized that cotreatment with lower doses of A-485 and JQ1 in combination could selectively inhibit synergism. To test this idea, we costimulated cells with IFNγ/TNFα along with A-485 and/or JQ1 at concentrations ranging from the IC_50_ or below and measured gene expression with NanoString to capture quantitative transcript counts. Existing methods for determining synergistic effects of drug combinations are not uniform. Web-based tools operate using arbitrary thresholds for synergism, and source code is not always publicly available to evaluate fitness in specific experimental models. To overcome these limitations, we developed and tested a general linearized model (GLM) to determine if the A-485 and JQ1 combinations synergistically inhibited chemokine induction. A Poisson regression model demonstrates that the multiplicative interaction term between A-485 and JQ1 successfully fits the data for *CXCL9* and *CXCL11*, but not for *CXCL10* (Figures 8A–8C; Table S2). This indicates that A-485 and JQ1 have a negative multiplicative effect on *CXCL9* and *-11* but not *-10*. Further examination of the model demonstrated that the negative effect observed with A-485 alone increases per experimental dose of JQ1 and vice versa. This is highlighted in Table S2, where, in the absence of A-485, *CXCL9* expression would be predicted to decrease by 3.5% per experimental dose of JQ1; however at 62 nM of A-485 the per experimental dose effect of JQ1 increased to 11.8%, demonstrating synergistic effect (Table S2). Based on this GLM analysis, both *CXCL9* and *CXCL11* demonstrated synergistic inhibition with A-485 and JQ1, while *CXCL10* was resistant to dual-inhibitor treatment at these lower, subIC50 concentrations (Figures 8A–8C).

Given the GLM results, we tested whether A-485 or JQ1 at or below IC_50_ could inhibit the production of secreted CXCL9, -10, and -11. HAECs were cotreated with IFNγ/TNFα and either A-485 or JQ1 for 8 h before supernatants were harvested and ELISAs was performed. These data revealed a strong trend toward synergistic inhibition as A-485 (62.5 nM) and JQ1 (15.6 nM) costimulation resulted in a 74.72% inhibition compared to A-485 (62.5 nM) alone (49.28% inhibition) and JQ1 (15.6 nM) alone (7.81% inhibition) (Figure 8D). CXCL10 was resistant to dual inhibition with A-485 and JQ1 (Figure 8E). Dual inhibition with A-485 and JQ1 did synergistically inhibit CXCL11 protein as A-485 (62.5 nM) and JQ1 (15.6 nM) costimulation resulted in a 59% reduction in protein levels, whereas A-485 (62.5 nM) alone or JQ1 (15.6 nM) alone resulted in 37% or no inhibition, respectively (Figure 8F). Together these data demonstrate that targeting p300/CBP and BET bromodomain proteins resulted in the inhibition of functional responses following IFNγ/TNFα costimulation. Importantly subIC_50_ concentrations were able to achieve specific inhibition of both transcript and protein levels for CXCL9 and CXCL11.

## Discussion

Transcriptional synergy—more-than-additive induction of a gene by two or more TFs—is an important mechanism for driving robust gene expression programs in development.^47^ In disease contexts, excess inflammation often arises via cytokine-mediated activation of signal-responsive TFs that dynamically induce proinflammatory programs. In particular, synergy elicited by IFNγ/TNFα is associated with severe SARS-CoV-2 illness.^2^ Here, we characterized the synergistic transcriptional response governed by IFNγ-STAT1 and TNFα-p65, as well as the changes in chromatin landscape and the chromatin coactivators involved.

Synergism arising from IFNγ and TNFα costimulation has previously been described in multiple cell types including ECs using candidate gene approaches.^2^^,^^8^ Here, unbiased RNA-seq identified that transcriptional synergism between IFNγ-STAT1 and TNFα−p65/NF-κB occurs rapidly within 1 h at a small subset of all cytokine-induced genes. Using stable isotope imaging with MIMS, we demonstrate that nascent RNA production is also detectable within 1 h in the nucleus, consistent with new transcription. Our outlier analysis classifying the chemokines *CXCL9*, *-10*, and *-11* as synergistic aligns with other studies that have observed similar induction in cultured HAECs, umbilical vein ECs, and cultured atherosclerotic plaques.^48^^,^^49^ Notably, we did not detect the synergistic induction of other previously reported genes such as *CCL5*, *IL-8*, and *HLA-A*.^8^^,^^9^^,^^13^ Compared to the 1-h duration presented here, these other studies stimulated cells with IFNγ/TNFα over longer periods of time, which could lead to secondary induction of other gene programs via action of TFs including IRFs. An additional explanation for the synergistic response is cytokine-induced changes in RNA stability that lead to increases in transcript counts, as previously shown for chemokine genes.^41^ No mRNA induction of RNA-binding proteins was evident by RNA-seq in response to IFNγ/TNFα stimulation (ELAVL1/HuR or LUBAC complex), and transcripts decreased significantly with cytokine washout. Our work focused on an early time point and primary transcriptional response to avoid some of these secondary effects. By first analyzing global transcriptomes with outlier analysis and not candidate genes, we identified that most genes in HAECs were induced to a similar degree by TNFα or combined IFNγ/TNFα, suggesting that TNFα responses dominate in ECs generally, as compared to IFNγ. However, the fact that synergistic transcription was restricted to a small fraction of all potential genes (*n* = 65) reinforces the concept that synergy is both tightly regulated and initially constrained.

One important concept in synergy is saturation. Studies of signal-responsive transcription commonly utilize high cytokine concentrations without defining the concentration-dependent effects on TF action vis-a-vis target gene output. Determination of the individual cytokine dose responses for IFNγ- and TNFα-regulated genes with NanoString enabled us to examine the thresholds for and magnitudes of transcriptional synergy even at non-saturating levels of STAT1 or p65 activity—as inferred from the maximal gene expression induced by IFNγ or TNFα, respectively. We found that synergy still occurs, albeit to a lesser degree, even at lower cytokine concentrations. Signaling crosstalk at the level of kinase cascades emanating from each receptor does exist between IFNγ and TNFα, which could impact TF activation in nuanced ways. We did not detect any evidence for crosstalk at the level of nuclear translocation, as IFNγ and TNFα only induced translocation of STAT1 and p65, respectively. However, more subtle changes in phosphorylation or other posttranslational modifications could impact DNA binding, transactivation, or coactivator interactions, leading to greater gene induction even at lower cytokine concentrations. IFNγ has also previously been shown to change looping at the *CXCL9*, *-10*, and *-11* locus in murine macrophages.^50^ Thus, it is possible that changes in chromatin confirmation driven by IFNγ can bring STAT1- and p65-regulated enhancers into closer physical proximity, amplifying the transactivation activity and inducing the synergistic response observed. Overall, the data establish that transcriptional synergy requires STAT1 and p65 and occurs across a range of their full transactivation potential.

Cooperativity is a prevailing model used to explain how TFs drive transcriptional synergy. Indeed, prior work dissecting a *CXCL9* promoter fragment with heterologous reporter constructs demonstrated that the number, orientation, and spacing of STAT1 and p65 binding sites have direct consequences on transactivation.^7^^,^^51^ While these prior data clearly support the cooperativity model, the focus on the promoter and lack of chromatinized DNA or genomic context limited the generalizability. Our paired ATAC-seq and TF CUT&RUN datasets suggest that cooperative binding does occur at the endogenous *CXCL9*, *-10*, *and -11* locus, as we identified multiple STAT1 and p65 motifs in accessible chromatin that were co-bound by both TFs. Several of these binding events occurred at intergenic regions, representing previously unrecognized distal enhancers. Furthermore, recruitment of p65 was significantly reduced in STAT1 KO cells, but not vice versa, suggesting a possible example of dynamic STAT1-assisted loading of p65, which has been described with inflammatory signaling in the hepatic acute phase response.^52^^,^^53^ In that context, p65 activation primed hepatocytes for STAT3 binding. Loss of p65 DNA occupancy due to increased STAT1 acetylation has been reported^54^; STAT1 can also interact directly with p300/CBP, which in turn acetylates p65 and thereby enhances p65 nuclear signaling. This hypothesis could explain why loss of STAT1 alters DNA binding of p65 but requires additional experimental validation in our system.^21^^,^^55^ Overall, the work herein identifies novel p65 and STAT1 functional interactions that drive synergistic gene induction in ECs.

A kinetic model of transcription has also been proposed to explain synergism. Two TFs acting on different steps of the transcription cycle (e.g., initiation and elongation) can promote dynamic and greater than multiplicative increases in Pol II processivity.^15^^,^^56^ Kinetic and cooperativity models are not mutually exclusive, and the contribution of each to synergy is difficult to deconstruct in endogenous gene contexts. However, a key prediction from both models is that chromatin-dependent signaling to Pol II via transcriptional coactivators plays a strong role. Yet, it was not clear at the outset of this study whether synergy genes might be especially resilient to coactivator disruption. The targeted inhibitor screen of coregulators identified selective vulnerabilities to p300/CBP and BET bromodomain inhibition. The selectivity of p300/CBP was evident targeting not only KAT function (A-485), but also the bromodomain (GNE-781) and p300/CBP degradation (dCBP). These results implicate coactivator recruitment, physical scaffolding, and acetylation by p300/CBP all as important determinants of synergy at the chemokine locus. In preclinical models, BET bromodomain inhibitors reduced inflammation associated with sepsis and cytokine storm.^29^^,^^57^ The role of BRD4 in control of transcriptional pause release via recruitment of the P-TEFb complex is well established and represents an important general mechanism for how BET bromodomain inhibitors disrupt signal-dependent pathologic transcription.^58^^,^^59^^,^^60^ More recent evidence also indicates that BRD4 inhibition alters cytokine-induced recruitment of Pol II to the *CXCL10* promoter in macrophages, suggesting that BRD4 can contribute to transcriptional initiation in certain signaling contexts as well.^61^ Furthermore, p300/CBP-dependent acetylation of p65 recruits BRD4 to TFs and histones via its bromodomains, leading to proinflammatory gene induction.^22^ SE-associated genes are also vulnerable to BET bromodomain inhibition.^23^ But, we did not detect differences in ATAC fragment size or signal, suggesting dynamic SE formation does not explain our results. One limitation is that H3K27-acetyl or BRD4 genome-wide profiling is needed to make this determination definitively. Neither p300/CBP nor BET bromodomain inhibition affected recruitment of STAT1 or p65. As such, the underlying mechanisms for inhibiting transcriptional synergy are likely stemming specifically from coactivator-dependent signaling from the TFs to the transcriptional machinery. When considered in totality, the known convergence of p300/CBP and BET bromodomain proteins on STAT1 and p65 pathways provides several candidate mechanisms to explain the sensitivity of synergy genes to each of these coactivators.

Dual p300/CBP and BET bromodomain inhibitors are currently in clinical development for cancer therapeutics.^28^ The rationale for this approach is, in part, based on the crosstalk between histone acetyltransferases and BET proteins. Here, we developed and tested a GLM framework to statistically determine if two inhibitors display synergistic inhibition on gene induction. This statistical approach provides rigor to data analysis when considering combination inhibitor therapies. Using a GLM identified that the combination of low doses (IC_50_ or lower) of p300/CBP (A-485) and BET bromodomain (JQ1) inhibitors can synergize to achieve inhibition of proinflammatory genes. At the protein level, these combinations also inhibited CXCL9 and CXCL11 protein secretion but had no impact on CXCL10. The reasons for resistance of CXCL10 protein to low-dose dual inhibition are unclear. Overall, given that IFNγ-STAT1 and TNFα−p65 signaling are implicated in acute and chronic diseases of inflammation (e.g., psoriasis, atherosclerosis, and cytokine storm syndromes including SARS-CoV-2), the transcriptional vulnerabilities identified herein suggest that low-dose inhibitor therapies could be a strategy for selectively targeting proinflammatory pathways, while avoiding global gene dysregulation and dose-limiting toxicities.

### Limitations of the study

All experiments were performed using immortalized ECs which were grown in static tissue culture conditions, differing from their native environment of laminar flow and shear stress. It remains unclear if the p65 and STAT1 binding observed in our CUT&RUN experiments uniquely occurs in the combined cytokine condition since we did not perform CUT&RUN on cells stimulated with individual cytokines. In addition, our work in cell culture may not fully capture the transcriptional responses that occur *in vivo*. Lastly, transcriptional coactivator binding profiles were not done in this index study. Occupancy maps of p300/CBP and individual BET bromodomain proteins will likely shed new light on underlying mechanisms controlling transcriptional synergy.

## STAR★Methods

### Key resources table

**Table:** 

REAGENT or RESOURCE	SOURCE	IDENTIFIER
**Antibodies**		

p65 (Rabbit)	Active Motif (39369)	RRID:AB_2793231
Beta Actin Mouse (Monoclonal)	ThermoFisher (Clone 15G5A/E2) (MA1-140) (Lot: TK276375)	RRID:AB_2536844
STAT1 (Rabbit)	Cell Signaling Technology (14995) (Lot:4)	RRID:AB_2716280
Goat anti-Rabbit Alexa Fluor™ 594	Thermo Fisher Scientific (A-11037) (Lot: 1777945)	RRID:AB_2534095
Goat anti-mouse Alexa Fluor™ 488	Thermo Fisher Scientific (A-21121) (Lot: 1704461)	RRID:AB_2535764
Rabbit IgG	Diagenode (C15410206) (Lot: RIG001AP )	RRID:AB_2722554
Rabbit monoclonal p65 (D14E12)	Cell Signaling Technology (8242) (Lot: 8242S)	RRID:AB_10859369
Rabbit monoclonal STAT1 (D4Y6Z)	Cell Signaling Technology (14995) (Lot: 4)	RRID:AB_2716280
Goat anti-Rabbit Alexa Fluor™ 647	ThermoFisher (A-21244) (Lot: 2247991)	RRID:AB_2535812
Mouse anti-FLAG antibody M2	Sigma Aldrich (F3165) (Lot: SLCJ3741 )	RRID:AB_259529
Mouse IgG	Diaganode (C15400001)	RRID:AB_2722553

**Chemicals, peptides, and recombinant proteins**		

Interferon Gamma (IFNγ)	PeproTech	300–02
Tumor Necrosis Factor Alpha (TNFα)	PeproTech	300-01A-100UG
BAY 11-7085	Med Chem Express	HY-10257
THZ1	Med Chem Express	HY-80013
Triptolide	Med Chem Express	HY-32735
Flavopiridol	Med Chem Express	HY-10005
BMS-345541	Med Chem Express	HY-10519
THZ531	Med Chem Express	HY-103618
Ruxolitinib	Med Chem Express	HY-50856
Vorinostat	Med Chem Express	HY-10221
Trichostatin A	Med Chem Express	HY-15144
dBET6	Med Chem Express	HY-112588
YKL-5-124	Med Chem Express	HY-101257
dCBP-1	Med Chem Express	HY-134582
GNE-781	Med Chem Express	HY-108696
JQ1	Med Chem Express	HY-13030
I-BET151	Med Chem Express	HY-13235
iBET-BD2	Med Chem Express	HY-136571
α-Amanitin	Med Chem Express	HY-19610
Mocetinostat	Med Chem Express	HY-12164
JIB-04	Med Chem Express	HY-13953
I-CBP112	Med Chem Express	HY-19541
iBET-BD2	Med Chem Express	HY-136571
A-366	Med Chem Express	HY-12583
GSK-J1	Med Chem Express	HY-15648
GSK-J4	Med Chem Express	HY-15648B
Cycloheximide	Sigma Aldrich	C4859
A-485	Med Chem Express	HY-107455
AU-15330	Med Chem Express	HY-145388
FHT 2344	Tocris Biotechne	7644/2
AltR S.p. HiFi Cas9 Nuclease V3	IDT	1081061
^13^C-Thymidine	Cambridge Isotope Laboratories	CLM-3647-PK
^15^N-Uridine	Cambridge Isotope Laboratories	NLM-812-PK
Biotin-conjugated Concanavalin A	Sigma Aldrich	C2272
Myone Streptavidin T1 DynaBeads	ThermoFisher	65601

**Critical commercial assays**		

Illumina Tagment DNA TDE1 Enzyme and Buffer Kit	Illumina	20034197
Human CXCL9/MIG Quantikine ELISA Kit	Bio-Techne	DCX900
Human CXCL10/IP -10 Quantikine ELISA Kit	Bio-Techne	DIP100
Human CXCL11/I-TAC Quantikine ELISA Kit	Bio-Techne	DCX110
nCounter Standard Master Kit	NanoString	NAA-AKIT-192
nCounter Standard Prep Pack	NanoString	NAA-PPCK-048
nCounter Standard Prep Plates	NanoString	NAA-PPLT-048
nCounter Standard Cartridges	NanoString	NAA-CART-048
8 well chamber slides	Chemglass Life Sciences	CGN-3530-008
Vascular Cell Basal Medium	ATCC	PCS-100-030
Endothelial Cell Growth Kit-VEGF	ATCC	PCS-100-041
ProLong Gold antifade reagent with DAPI	Invitrogen	P36931 Lot: 2501098
Cell Proliferation Kit I (MTT)	Roche	11465007001
Pure-link RNA Mini kit	Invitrogen, ThermoFisher	12183025
NEBNext® Poly(A) mRNA Magnetic Isolation Module	NEB	E7490
NuPage 4–12% Bis Tris 12 well gels	Invitrogen	NP0322BOX
Kaleidoscope Protein ladder	BioRad	1610375
DNA Clean & Concentrator-5	Zymo Research	D4004
Fugene HD	Fugene	HD-1000
TransDux Max	Systems Biosciences	LV860A-1 Lot:230331-003
PEG-IT Virus Precipitation solution	Systems Biosciences	LV825A-1
Universal Mycoplasma Detection Kit	ATCC	30-1012K
iScript RT-qPCR Sample Prep Reagent	Bio-Rad	1708899
iTaq™ Universal SYBR® Green One-Step Kit	BioRad	1725150

**Deposited data**		

ATAC-seq, CUT&RUN, and RNA-seq data	NCBI GEO	GSE232168

**Experimental models: Cell lines**		

TeloHAEC	ATCC (CRL-4052)	RRID:CVCL_Z065
293T/17	ATCC (CRL-11268)	RRID:CVCL_1926

**Oligonucleotides**		

STAT1 cRNA (AGTGGTTAGAAAAGCAAGAC )	IDT	NA
STAT1 cRNA (TTCCCTATAGGATGTCTCAG )	IDT	NA
p65 cRNA (AGCGCCCCTCGCACTTGTAG )	IDT	NA
Alt-R CRISPR-Cas9 Negative Control crRNA	IDT	1072544
Alt-R CRISPR-Cas9 tracrRNA	IDT	1073191
CXCL9 For PCR Primer (GCTGGTTCTGATTGGAGTGC )	IDT	NA
CXCL9 Rev PCR Primer (GAACAGCGACCCTTTCTCAC )	IDT	NA
CXCL10 For PCR Primer (CTGTACGCTGTACCTGCATCA )	IDT	NA
CXCL10 Rev PCR Primer (CAACACGTGGACAAAATTGG )	IDT	NA
CXCL11 For PCR Primer (GAGTGTGAAGGGCATGGCTA )	IDT	NA
CXCL11 Rev PCR Primer (GGGGAAGCCTTGAACAACTG )	IDT	NA
SELE For PCR Primer (GAGGTGCAGCAAGAAGAAGCTTCG )	IDT	NA
SELE Rev PCR Primer (CACTGAAGCCAGGGTCACAC )	IDT	NA
Nol7 For PCR Primer (CGGAAGCGAGAGAAGAGGAG )	IDT	NA
Nol7 Rev PCR Primer (CGCTTCCTCTTCTCCTTCAG )	IDT	NA
HPRT1 For PCR Primer (CTTTGCTGACCTGCTGGA )	IDT	NA
HPRT1 Rev PCR Primer (TGTCCCCTGTTGACTGGT )	IDT	NA

**Software and algorithms**		

Bowtie2	Langmead et al.^62^	NA
HOMER	Heinz et al.^63^	NA
MACS2	Zhang et al.^64^	NA
DESeq2	Love et al.^65^	NA
Custom NanoString Analysis Code	This study	https://github.com/jonathanbrown484/synergy
Custom GLM Code	This study	https://github.com/jonathanbrown484/synergy

**Other**		

3xFLAG-STAT1 Plasmid	Addgene	220323
3xFLAG-RELA Plasmid	Addgene	220324

### Resource availability

#### Lead contact

Further information and requests for resources and reagents should be directed to and will be fulfilled by the lead contact, Jonathan D. Brown (jonathan.d.brown@vumc.org).

#### Materials availability

Plasmids generated in this study have been deposited to Addgene (3xFLAG-STAT1 = 220323, 3xFLAG-RELA = 220324).

#### Data and code availability

• ATAC-sequencing, CUT&RUN, and RNA-sequencing datasets generated in this manuscript have been deposited at NCBI GEO under the accession number: GSE232168 and are publicly available.
• Custom NanoString analysis code and GLM for determining synergistic inhibition are publicly available at Github:/jonathanbrown484/synergy
• Any additional information required to reanalyze the data reported in this work paper is available from the lead contact upon request.

### Experimental model and study participant details

H-TERT-immortalized human aortic endothelial cells (TeloHAEC) (ATCC: CRL-4052) (RRID:CVCL_Z065) were purchased from ATCC. These cells were extracted from a 23-year old female donor and have been immortalized via stable expression of the human Telomerase catalytic subunit hTERT. No information about race or ancestry is provided by ATCC. Cells were tested for mycoplasma contamination using the Universal Mycoplasma Detection Kit (ATCC: 30-1012K).

### Method details

#### ATAC-seq experimental

Cells were treated with media lacking hydrocortisone for 16–20 h before stimulation. Cells were unstimulated or stimulated for 1 h IFNγ, TNFα, or IFNγ/TNFα (*n* = 2 per condition) before cells were lifted from the plate. 50,000 cells were used to perform ATAC-seq as previously described.^66^ 0.1% BSA was added to cells to assist with pelleting.

##### ATAC-seq computational

ATAC-seq fastq files were processed using the standard ENCODE pipeline. Reads were filtered to contain reads which were less than 150bp to obtain information from nucleosomal free reads (NFRs).

##### ATAC-seq differential peak calling

To make comparisons across 4 conditions the following was performed: First, BAM files produced by the ENCODE pipeline were converted to SAM files using samtools view -h -o output.sam input.bam.^67^ Tag directories were produced for each replicate using the makeTagDirectory function from the HOMER package.^63^ For each set of replicates, the tag directories were merged producing one tag directory per condition.^63^ Differential peaks were called using IFNγ/TNFα peaks, desired tag directories for each desired pairwise comparison using the getDifferentialPeaks function from HOMER with F- 1.5.^63^ The function annotatePeaks.pl from HOMER was used to create an annotated normalized signal matrix for each differential comparison with options ‘hg38’, ‘-tbp 1’, ‘-rpkm’, and ‘-strand both’.^63^ To obtain peaks that are unique to the IFNγ/TNFα condition specifically, pairwise comparisons between IFNγ/TNFα and the three other conditions (unstimulated, IFNγ, or TNFα) were performed. The results were then filtered to peaks that reached a *p*-value of less than 0.0001in all three comparisons, which indicates higher accessibility in the synergy condition compared to all other treatment conditions.

##### ATAC-seq motif signal plots

Localization of p65 and STAT1 motifs relative to the summit of each peak was determined using annotatePeaks.pl peakfile.txt hg38 -size 150 -hist 15 -m p65.motif STAT1.motif.^63^ The resultant histogram was utilized to produce line graphs. ATAC-seq signal plots for IFNγ/TNFα+DMSO (Vehicle), IFNγ/TNFα+A-485, and IFNγ/TNFα+JQ1 were produced using annotatePeaks.pl -size 2000 -hist 10 -d tag_directories > out_file.^63^

##### ATAC-seq motif enrichment

bed2fasta function from MEMEsuite was utilized to produce fasta files from BED4 formatted peak files.^68^ AME from MEMEsuite was utilized to determine motif enrichment in peaks that are unique in the IFNγ/TNFα condition using the following script ame -control --shuffle-- --oc output --method ranksum --scoring avg fasta_file.fasta consensus_pwms.meme.^28^^,^^69^^,^^70^ Families of motifs were utilized instead of individual motifs to avoid representing and performing statistical analysis on redundant motifs. Families of motifs were classified via non-redundant TF motif clustering as previously described.^70^

##### ATAC-seq peak size, signal, and normalized signal

Peaks were binned by the genomic annotation provided by the annotatePeaks command in HOMER.^63^ Peaks with the intergenic annotation were considered distal elements with all other annotations binned as gene body. Peak size and signal were obtained from the annotated peak files and utilized to generate the normalized signal which is signal/peak size.

#### Cell culture

hTERT-immortalized human aortic endothelial (TeloHAEC) (ATCC: CRL-4052) (RRID:CVCL_Z065) cells were subcultured using Vascular Cell Basal Medium (ATCC: PCS-100-030) supplemented with Endothelial Cell Growth Kit-VEGF (ATCC: PCS-100-041). Cells were cultured until p25 and media was changed every 3 days. Cells were changed into media lacking hydrocortisone 16–20 h before stimulation.

#### Cell viability experiment

200,000 cells were plated in media lacking hydrocortisone. 16 h post plating they were stimulated with 50 ng/mL IFNγ and 25 ng/mL TNFα for 48 h (*n* = 3 per condition). Cells were then harvested and cell viability was assessed via staining with Trypan Blue and the total number of viable and dead cells were counted.

#### CRIPSR KO generation

crRNAs were designed for *RELA* and *STAT1* using the CRISPOR tool.^71^ Ribonucleoprotein (RNP) assembly and CRISPR knockout was performed as previously described.^72^ In brief crRNA and tracrRNA were mixed 1:1 and incubated at 95°C for 5 min then cooled at RT for 10 min before AltR S.p. HiFi Cas9 Nuclease V3 (IDT 1081061) was added to gRNA. The RNP complex was incubated at 30°C for 20 min, then electroporated into HAECs using the NEON Electroporation system with settings: 1500V, 20 ms pulse width x 2 pulses. Cells were electroporated with RNPs containing 2 gRNAs (for STAT1), and 1 gRNA (for p65), or 1 non-target gRNA (see key resources table for sequences). Knockout of STAT1 and/or p65 was confirmed via Western Blotting.

#### Cloning and lentivirus production

To clone 3xFLAG-STAT1 and 3xFLAG-p65 constructs into lentivirus gateway transfer vector we synthesized gBlocks (IDT) with attB sites and a stop codon at the end of each TF cDNA reading frame. We performed BP and LR cloning per manufacturer protocol (Gateway Cloning ThermoFisher) using pDONR221 for the donor vector and pLEX305-C-dTAG (Addgene #91798) as the destination vector. Of note, the destination vector first required that the antibiotic selection be changed from Puromycin to Blasticidin (BSD gene), because TELO-HAECs have a puromycin resistance gene present due to expression of Telomerase used to immortalize the cells. We changed Puromycin to BSD by restriction enzyme cloning with a gBlock containing compatible KpnI and HpaI sites flanking the BSD gene. After gateway cloning the 3xFLAG constructs, we transformed NEB10-beta cells at 30°C for 24 h. Clones were sequence verified. For lentivirus production, 293T/17 cells were transfected with 20 μg of the transfer plasmid, 15 μg of psPAX2 packaging plasmid (addgene 12260) and 5 μg of pMD2.G envelope plasmid (addgene 12259) with Fugene HD (3:1 ratio) in high glucose DMEM with 10% FBS with no antibiotics. 48 h after transfection supernatant was harvested, dead cells cleared with centrifugation at 2000 rpm and then filtered with 0.45 micron syringe filter. Virus was precipitated with PEG-IT (Systems Biosciences) per manufacturer protocol for 16 h at 4°C, concentrated 100-fold in sterile PBS and then aliquoted for storage at −80. For infection of HAECs, 10 μL of virus were used, based on cell titration studies of p65 and STAT1 protein expression. Cells were selected with blasticidin (3 μg/mL) for 7–10 days prior to validation studies and experiments.

#### CUT&RUN

CUT&RUN was performed with a few modifications for transcription factor enrichment as previously described.^73^ In brief, adherent ECs were trypsinized, counted and 1e6 cells per IP were fixed in 0.1% formaldehyde for 1 min then quenched with 125 mM glycine for 1 min at room temperature. Cells were bound to 10uL of activated concanavalin A conjugated streptavidin (MyOne T1, Thermo 65601) beads per IP as previously described.^74^ Beads were washed with wash buffer containing (20mM HEPES pH 7.5, 150mM NaCl, 0.5mM Spermidine, 0.1% BSA Protease and Phosphatase Inhibitors and 1% BSA). Cells were permeabilized in Wash buffer supplemented with 0.01% digitonin, 0.1% Tween and 0.1% NP-40 for 10 min. After permeabilization, immunoprecipitation was performed with 0.7 μg of antibody per IP in Digitionin-Wash buffer supplemented with 2mM EDTA. Samples were incubated overnight on a nutator at 4°C. Samples next washed in the Dig-wash buffer twice and then incubated with pAG-MNase (MOLOX, 700 ng/mL) for 1 h at 4°C to bind pAG-MNase, then washed with Dig-wash buffer twice. DNA Cleavage reaction was done with 100mM CaCL_2_ for 30 min while samples were submerged in ice. Cleavage was terminated with STOP buffer with EDTA/EGTA and chromatin released by gentle heating of samples at 37°C for 30 min. Samples were decrosslinked with 1% SDS and 250 μg/mL proteinase K for 4 h in a thermomixer at 65°C. DNA was then purified via phenol-chloroform-isoamyl alcohol extraction and Ethanol precipitation. Sequencing libraries were generated using the NEBNext Ultra II DNA Library Prep Kit (NEB #E7645) with transcription factor modifications.^73^ PCR program to enrich for short fragments as follows: Step 1: 45 s at 98°C. Step 2: 15 s at 98°C. Step 3: 10 s at 60°C. Step 4: Repeat Steps 2–3 for a total of 14 cycles. Step 5: 1 min at 72°C final extension.

#### CUT&RUN computational

Reads were trimmed using trimmomatic and then mapped to hg38 using bowtie2. Duplicate reads were discarded and peaks were called using HOMER as previously described.^63^ In short peaks were called using the IFNγ/TNFα + Veh peaks and tag directories for each desired pairwise comparison. Peaks were called using getDifferentialPeaks – IFNγ/TNFα + Veh peaks -d tag directories. Motif enrichment was performed as described above for ATAC-seq.

#### Difference and outlier detection

To identify potentially non-additive effects of joint treatment, for each gene differentially expressed between IFNγ/TNFα-treated group and untreated group, the fold change difference was calculated by the following approach. Firstly, we calculated the log_2_(fold change) between the IFNγ/TNFα-treated group and the untreated group. Next, we computed the sum of the log_2_(fold changes) for the TNFα-treated group and the IFNγ-treated group, each compared to the untreated group. Both of the above results were used to calculate the combined difference value expressed as (IFNγ/TNFα – (TNFα + IFNγ)). Next, the outliers within these difference values were detected using the interquartile range (IQR) method. The criterion for classifying a value as an outlier was based on two conditions: either the difference value exceeded the highest quantile cutoff by 1.5 times the IQR or it fell below the lowest quantile cutoff by 1.5 times the IQR. The functions IQR and quantile are from the package stats included in R.

#### ELISAs

70,000 cells were plated into a 24 well plate in media lacking hydrocortisone for 16–20 h before stimulation. Cells were stimulated with cytokines or cytokines + inhibitors for 8 h before supernatant was harvested (*n* = 3 per condition). Supernatant was centrifuged for 5 min at 500g; supernatants were then stored at −80C until use. ELISAs were performed per manufacturer’s instructions (CXCL9: Bio-techne (DCX900) CXCL10: Bio-techne (DIP100) CXCL11: Bio-techne (DCX110)). All supernatants for CXCL9 and CXCL11 were run undiluted. Supernatants for CXCL10 in the IFNγ/TNFα, IFNγ/TNFα +DMSO, IFNγ/TNFα +62nM A-485, IFNγ/TNFα +15.6nM JQ1, IFNγ/TNFα +62nM A-485 15.6nM JQ1, IFNγ/TNFα +31nM A-485, IFNγ/TNFα +7.8nM JQ1, and IFNγ/TNFα +31nM A-485 7.8nM JQ1 conditions were diluted 1:240. Supernatants for CXCL10 in the IFNγ/TNFα +2000nM A-485 and IFNγ/TNFα +500nM JQ1 were diluted 1:120.

##### GLM computational

NanoString data was processed as described below. The averaged normalized transcript counts were utilized to generate a general linearized model (GLM) utilizing a Poisson regression model. This GLM included variables to examine the ability of A-485 alone, JQ1 alone or the multiplicative interaction between both inhibitors to explain the observed experimental data. The ability of these models to fit the experimental data was assessed via an ANOVA using Wald statistics. Estimated marginal means utilizing the multiplicative interaction term were calculated using the emmeans package in R for each gene. Trends at each dose of A-485 or JQ1 were calculated using emmtrends in R on the output from the emmeans function described above.

##### Hill coefficient modeling and IC_50_ calculations

Hill coefficient modeling and subsequent IC_50_ calculations were performed using the hillfit python package with the bottom_param = False and log_x = True.

#### Immunofluorescence

30,000 cells were plated into 8-well chamber slides (Chemglass Life Sciences CGN-3530-008). Cells were incubated in media lacking hydrocortisone for 16–20 h before being stimulated for 5, 15, 60, or 240 min. Cells were fixed with 4% formaldehyde for 15 min at RT and washed 3x with PBS. Cells were permeabilized for 10 min with methanol at −20C, washed 3x with PBS and blocked for 1 h at RT. Antibodies were added at the dilution of 1:6000, 1:1000, and 1:200 (IgG: Rabbit IgG Diagenode C15410206 Lot: RIG001AP RRID:AB_2722554, p65: Rabbit monoclonal p65 Cell Signaling Technology D14E12 Lot: 8242S RRID:AB_10859369 and STAT1: Rabbit monoclonal STAT1 Cell Signaling Technology 1495S Lot: 4 RRID:AB_2716280) respectively and antibodies were incubated overnight at 4C. Secondary antibody (ThermoFisher Goat anti-Rabbit Alexa Fluor 647 A21244 Lot: 2247991 RRID:AB_2535812) was added at a dilution of 1:500 and incubated for 1 h at RT. DAPI stain (ProLong Gold antifade reagent with DAPI Invitrogen: P36931 Lot: 2501098) was added to slides and cover slips were applied. Images were captured on Zeiss LSM880 Confocal Microscope 10X magnification. Scale bars indicate 100 micron.

#### Multi-isotope imaging mass spectrometry (MIMS)

Stable isotope tracers for DNA synthesis (^13^C-thymidine) and RNA synthesis (^15^N-uridine) were purchased from Cambridge Isotope Laboratories. HAEC were cultured in ^13^C-thymidine at a concentration of 50 (μM) for 14 days. At the final passage prior to the experiment, cells were seeded onto silicon wafers in culture dishes. ^15^N-uridine (50 μM) was added for 1 or 4 h with cytokines, then washed with PBS and fixed with 4% paraformaldehyde for 15 min. The cells were then washed with PBS, subjected to serial ethanol dehydration (50%, 70%, 90%, 95%, 100%), and air dried. Samples were then analyzed with a NanoSIMS 50L instrument (CAMECA), using previously published analytical methods.^38^^,^^39^^,^^75^ ^13^C-thymidine labeling was measured by the ^13^C^12^C^−^/^12^C_2_^−^ ratio and ^15^N-uridine was measured by the ^12^C^15^N^−^/^12^C^14^N^−^ ratio as described previously.^38^^,^^39^^,^^75^ The instrument was also tuned to capture ^32^S^−^. Image files were visualized and analyzed with a custom plugin to ImageJ: OpenMIMS 3.0: https://github.com/BWHCNI/OpenMIMS.^75^ ^32^S^−^ and ^13^C^12^C^−^/^12^C_2_^−^ ratio images were used to guide manual selection of regions of interest (ROIs) corresponding to the entire area of the nucleus and the cytoplasm (excluding the nucleus). The corresponding isotope ratios were then extracted representing the mean of all pixels contained within each respective ROI. Isotope ratio data are displayed in the manuscript (Figure 1) as hue saturation intensity (HSI) images. The lower bound of the scale (blue) was set at natural background (e.g., for ^15^N-uridine data a lower bound of 0 is equivalent to the natural background of 0.37% = no labeling and an upper bound of 100 corresponds to a ratio of 0.74%). For representative images, the upper bound of the scale was set to demonstrate regional differences in labeling. Importantly, the underlying quantitative data are unmodified by changes in the scaling of displayed images.

#### MTT assay

5,000 cells were plated into a 96 well plate in media lacking hydrocortisone (*n* = 3 per condition). MTT assay was performed per manufacturer’s instructions (Cell Proliferation Kit I (MTT) Roche 11465007001). In brief cells were stimulated for 48 h before MTT assay labeling reagent was added. MTT assay labeling reagent incubated for 4 h at 37C and 5% CO2 after which MTT solubilization reagent was added. Plates were allowed to incubate overnight before absorption was measured at 550nm.

#### NanoString experimental

20,000 cells were plated into 96 well plate in media lacking hydrocortisone 16–20 h before stimulation. Cells were stimulated for 1 h before they were harvested with iScript RT-qPCR Sample Prep Reagent (Bio-Rad 1708899). Lysates were transferred to PCR strips and placed at −80C or processed immediately after harvesting. NanoString hybridization was performed per manufacturer’s instructions with a custom probe set. In short A and B probes were resuspended at 5nM and 25nM respectively probes were then mixed and added to 7uL of sample lysate. Samples hybridized at 67°C for 24 h. Samples were quick spun down and then pooled and loaded on the NanoString nCounter Max prep station.

##### NanoString computational

The output of each.RCC file was processed to produce a file containing transcript counts for each gene of interest mapped to the position in the 96 well plate. The data was normalized in the following manner: first differences in hybridization were normalized by generating the geometric mean of the positive control samples within each column. The geometric mean was divided by the positive control of each well to generate the scaling factor which was applied to all genes in in the corresponding well, this was repeated to normalize all samples in a given column after which the geometric mean was calculated for the next column and the process was repeated again.

Next the samples were normalized to account for plex set hybridization variability by generating the geometric mean for each gene in the calibration column. A scaling factor was generated by multiplying the geometric mean of all calibration samples by 1 over the transcript count for a given gene in the calibration sample in a specific plex set. This scaling factor was applied to all transcript measurements in the specific plex set and was repeated for each gene that was measured and for all plex sets.

Housekeeper normalization was done by obtaining the geometric means of all the housekeeper genes at each position in the plex set. Next the arithmetic mean of all geometric means is calculated and used to generate a scaling factor dividing the arithmetic mean of all housekeeper geometric means by the geometric mean of all house keeper genes in a given position. This scaling factor is then applied to all transcripts in that corresponding position.

#### One step, real-time qPCR

10,000 TELOHAECs were plated in a 96 well plate containing media without hydrocortisone 16–20 h before stimulation. Cells were stimulated for 1 h before they were harvested with iScript RT-qPCR Sample Prep Reagent (Bio-Rad 1708899). qPCR was performed per manufacturer’s instructions using 1uL of lysate and the iTaq Universal SYBR Green One-Step Kit (Bio-Rad 1725151).

#### RNA-seq

75,000 cells were plated in media lacking hydrocortisone for 16–20 h. Cells were simulated for 1 h before cells were harvested using Pure-link lysis buffer containing 10% beta-mercaptoethanol. Lysate was processed immediately using the Pure-link RNA Mini kit (Invitrogen, ThermoFisher 12183025) or stored at −80C. RNA was isolated following the manufacturer’s instructions for the Pure-link RNA Mini kit (Invitrogen, ThermoFisher 12183025). RNA-seq libraries were generated using NEBNext Poly(A) mRNA Magnetic Isolation Module (NEB E7490) per manufacturer’s instructions. Paired end 150bp sequencing was performed on an Illumina NovaSeq 6000 machine targeting 20,000,000 reads per sample.

#### RNA-seq computational analysis

Reads were trimmed to remove adapter sequences using Cutadapt (v2.10)^76^ and aligned to the Gencode GRCh38.p13 genome using STAR (v2.7.8a).^77^ Gencode v38 gene annotations were provided to STAR to improve the accuracy of mapping. Quality control on both raw reads and adaptor-trimmed reads was performed using FastQC (v0.11.9) (www.bioinformatics.babraham.ac.uk/projects/fastqc). featureCounts (v2.0.2)^78^ was used to count the number of mapped reads to each gene. Heatmap3 was used for cluster analysis and visualization.^79^ Significantly differential expressed genes with absolute fold change ≥ 2 and FDR adjusted *p* value ≤ 0.05 were detected by DESeq2 (v1.30.1).^65^

#### Western Blot

200,000 TELO-HAECs were harvested and pelleted by centrifugation at 500*g* for 5 min at 4C. Cells were lysed via the addition of whole cell lysis buffer and passed through a 28-gauge insulin needle 10 times. Samples were rested on ice for 10 min before being centrifuged at 21,000g for 7 min at 4C. Supernatant was transferred to new tube and protein concentration determined via BCA assay. 22.5 μg of protein was loaded into NuPage 4–12% Bis Tris 12 well gels (Invitrogen NP0322BOX). Kaleidoscope Protein ladder (BioRad 1610375) was diluted 1:1 in 1x LDS and gel was run at 120V for 2 h in 1x MOPS buffer. Gel was transferred to a nitrocellulose membrane at 30V for 90 min in 10% methanol transfer buffer. After transfer the membrane was transferred to block for 1 h at RT on rocker. p65 blot was performed by diluting p65 (Rabbit Active Motif 39369 RRID:AB_2793231) 1:2500, beta actin mouse (Monoclonal (Clone 15G5A/E2) ThermoFisher MA1140 Lot: TK276375 RRID:AB_2536844) was diluted 1:2500. STAT1 blot was performed by diluting Rabbit STAT1 (Cell Signaling Technology 14995S Lot:4 RRID:AB_2716280) diluted 1:1000 and beta actin mouse (Monoclonal (Clone 15G5A/E2) ThermoFisher MA1140 Lot: TK276375 RRID:AB_2536844) was diluted 1:2500. Anti-FLAG antibody (Sigma Aldrich, F3165) was used at a final concentration of 1 μg/mL. Membranes were washed 3× in 0.1% TBS-T and secondary antibodies were diluted 1:1000 (Goat anti-Rabbit) and 1:1000 (Goat anti-mouse) and incubated for 1 h while rocking. After secondary antibody, membranes were again washed 3× in 0.1% TBST then imaged on LICOR Odyssey. Densitometry was performed using Image Studio Lite from LI-COR Biosciences.

### Quantification and statistical analysis

For assessment of statistical significance both R and Prism were utilize. Details on the statistical tests performed for individual experiments can be found in the figure legend of each respective figure. Any *p*-value less than 0.05 was determined to be statistically significant in this study. For RNA-seq and ATAC-seq adjusted *p*-values were used.

## Supplemental information

Document S1. Figures S1–S7 and Table S1

Table S2. Statistics for Poisson regression model and predicted per unit effects of inhibitors, related to Figure 8

Table S3. Differential genes RNA-seq, related to Figure 1

Table S4. Differential ATAC-seq peaks, related to Figures 5 and 7

Table S5. Differential CUT&RUN peaks, related to Figures 5 and 7
