# Supplementary material for: Transcriptional synergy in human aortic endothelial cells is vulnerable to combination p300/CBP and BET bromodomain inhibition
Source: iScience. 2024 May 16;27(6):110011. doi: 10.1016/j.isci.2024.110011 (PMC11167439; doi:10.1016/j.isci.2024.110011)
Supplement: Document S1. Figures S1–S7 and Table S1 [file mmc1.pdf]

iScience, Volume *27*

Supplemental information

Transcriptional synergy in human aortic endothelial cells is vulnerable to combination p300/CBP and BET bromodomain inhibition

Ronan C. Bracken, Lindsay M. Davison, Dennis P. Buehler, Maci E. Fulton, Emily E. Carson, Quanhu Sheng, Lindsey K. Stolze, Christelle Guillermier, Matthew L. Steinhauser, and Jonathan D. Brown

Figure S1

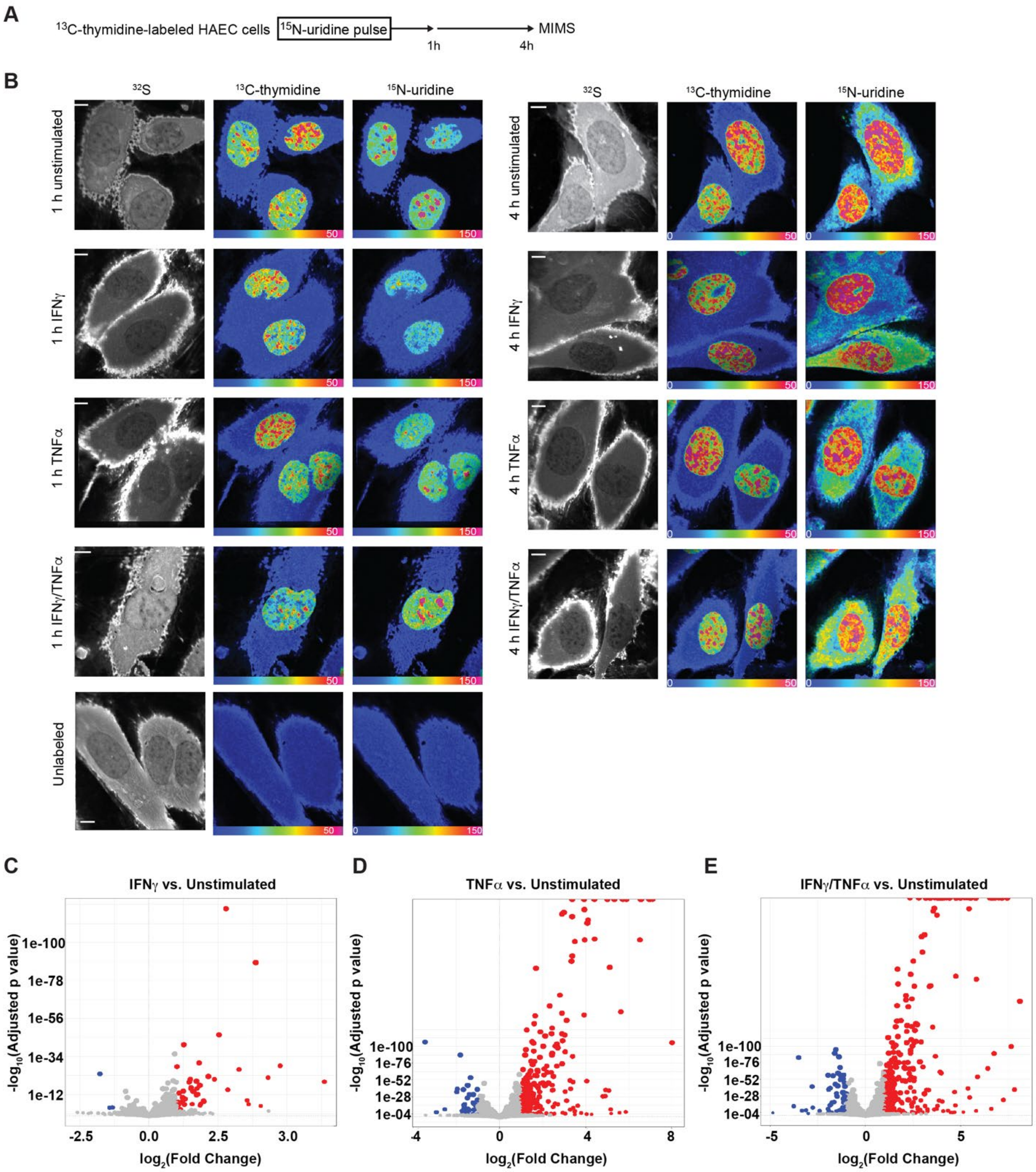

A
13C-thymidine-labeled HAEC cells
15N-uridine pulse
1h
4h
MIMS
B
32S
13C-thymidine
15N-uridine
1 h unstimulated
1 h IFNγ
1 h TNFα
1 h IFNγ/TNFα
Unlabeled
4 h unstimulated
4 h IFNγ
4 h TNFα
4 h IFNγ/TNFα
C
IFNγ vs. Unstimulated
D
TNFα vs. Unstimulated
E
IFNγ/TNFα vs. Unstimulated
-log10(Adjusted p value)
log2(Fold Change)

Figure S1 (related to Figure 1): IFNγ/TNFα costimulation of HAECs induces nascent RNA production and synergistic expression of proinflammatory chemokine genes

(A) Schematic of experimental design for MIMs experiments.

(B) MIMS images of unstimulated HAECs, HAECs stimulated with IFNγ (50ng/mL), TNFα (25ng/mL) or IFNγ/TNFα for one hour (left) or four hours (right). At the bottom is shown MIMS images of cells with no isotope. Quantitative ^{32}S images reveal cellular and nuclear contours (left column). Hue saturation intensity images are used to visually represent isotope ratio measurements and in turn map ^{13}C-thymidine labeling of DNA ($^{13}C/^{12}C$, middle) and ^{15}N-uridine labeling of nascent RNA ($^{15}N/^{14}N$, right). The lower bound of the color scale (blue) is set to natural background (^{13}C=1.1%; ^{15}N=0.37%) and the upper bound of the scale is set to reveal differential labeling and expressed as % above natural background.

(C) Volcano plot of $\log_2$(FC) vs. $\log_{10}$(Adjusted p-value) for differential genes in IFNγ stimulated vs. Unstimulated HAECs.

(D) Volcano plot of $\log_2$(FC) vs. $\log_{10}$(Adjusted p-value) for differential genes in TNFα stimulated vs. Unstimulated HAECs.

(E) Volcano plot of $\log_2$(FC) vs. $\log_{10}$(Adjusted p-value) for differential genes in IFNγ/TNFα stimulated vs. Unstimulated HAECs.

Figure S2

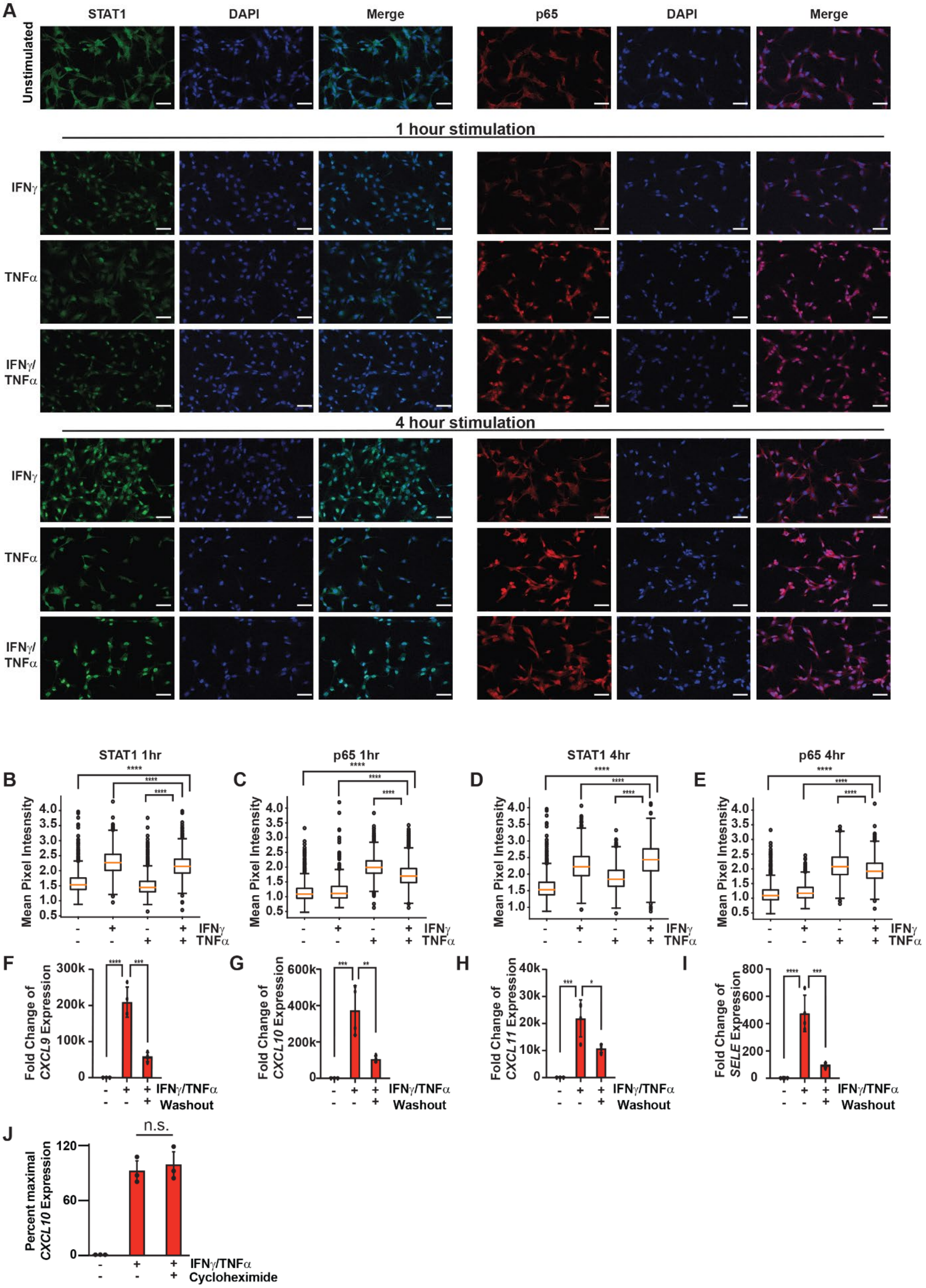

A
STAT1
DAPI
Merge
p65
DAPI
Merge
Unstimulated
1 hour stimulation
IFNγ
TNFα
IFNγ/ TNFα
4 hour stimulation
IFNγ
TNFα
IFNγ/ TNFα
B
STAT1 1hr
C
p65 1hr
D
STAT1 4hr
E
p65 4hr
Mean Pixel Intesnsity
IFNγ
TNFα
F
Fold Change of CXCL9 Expression
G
Fold Change of CXCL10 Expression
H
Fold Change of CXCL11 Expression
I
Fold Change of SELE Expression
IFNγ/TNFα
Washout
J
n.s.
Percent maximal CXCL10 Expression
IFNγ/TNFα
Cycloheximide

Figure S2 (related to Figure 2): Transcriptional synergy elicited by IFNγ/TNFα signaling requires STAT1 and p65

(A) Representative photomicrographs of STAT1 (green) and p65 (red) immunofluorescence in TeloHAECs treated with vehicle or cytokines for 1 hour or 4 hours. Nuclei are indicated by DAPI staining (blue color). All samples were imaged on a Zeiss LSM880 Confocal Microscope with 10X objective. Scale bars indicate 100 μm.

(B-E) Box and whisker plots of mean fluorescence intensity for nuclei measured in (A). 1 hour STAT and p65 in (B-C) and 4 hour STAT and p65 in (D-E). Kruskal-Wallis test was performed followed by a pairwise Wilcoxon test to determine statistical differences between treatment groups. ***p-value < 0.001; **** p-value < 0.0001.

(F-I) Bar graphs of mRNA expression of *CXCL9 (F), CXCL10 (G), CXCL11 (H) or SELE (I)* in cells after cytokine stimulation (1 hour) followed by cytokine washout for 3 hours. mRNA shown as fold change compared to unstimulated baseline. One-way ANOVA was performed followed by Tukey's post-hoc analysis to determine statistical differences between treatment groups (n.s. = not significant). * p-value < 0.05; ** p-value < 0.01 *** p-value < 0.001; **** p-value < 0.0001.

(J) mRNA expression in HAECs treated with or without Cycloheximide (100 μg/mL) and IFNγ/TNFα (1 hour) versus no cytokine (n=3 per condition)**.** One-way ANOVA was performed followed by Tukey's post-hoc analysis to determine statistical differences between treatment groups (n.s. = not significant).

Figure S3

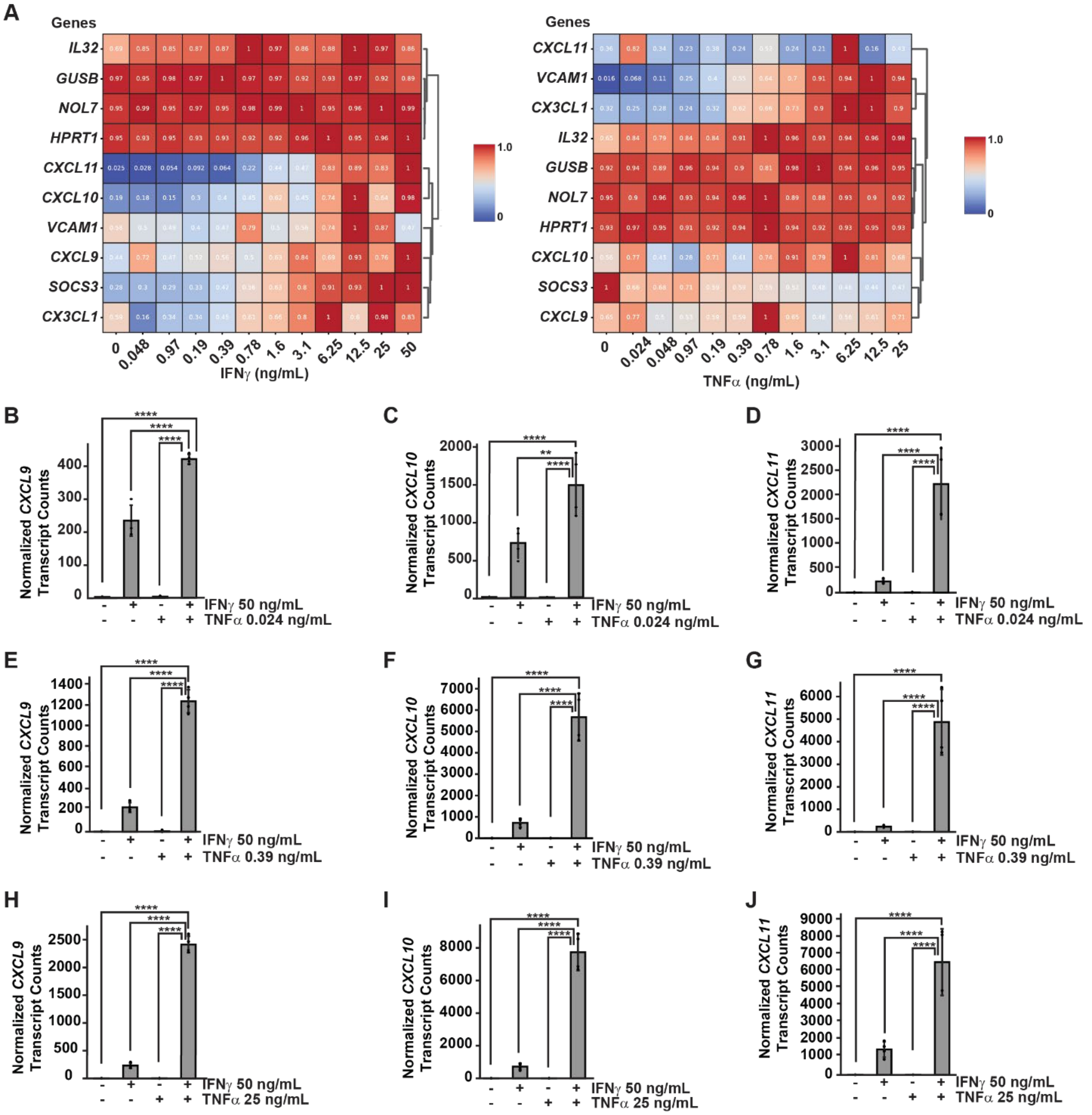

A
Genes
IL32
GUSB
NOL7
HPRT1
CXCL11
CXCL10
VCAM1
CXCL9
SOCS3
CX3CL1
1.0
0
IFNγ (ng/mL)
0
0.048
0.97
0.19
0.39
0.78
1.6
3.1
6.25
12.5
25
50
Genes
CXCL11
VCAM1
CX3CL1
IL32
GUSB
NOL7
HPRT1
CXCL10
SOCS3
CXCL9
TNFα (ng/mL)
0
0.024
0.048
0.97
0.19
0.39
0.78
1.6
3.1
6.25
12.5
25
B
Normalized CXCL9 Transcript Counts
IFNγ 50 ng/mL
TNFα 0.024 ng/mL
C
Normalized CXCL10 Transcript Counts
IFNγ 50 ng/mL
TNFα 0.024 ng/mL
D
Normalized CXCL11 Transcript Counts
IFNγ 50 ng/mL
TNFα 0.024 ng/mL
E
Normalized CXCL9 Transcript Counts
IFNγ 50 ng/mL
TNFα 0.39 ng/mL
F
Normalized CXCL10 Transcript Counts
IFNγ 50 ng/mL
TNFα 0.39 ng/mL
G
Normalized CXCL11 Transcript Counts
IFNγ 50 ng/mL
TNFα 0.39 ng/mL
H
Normalized CXCL9 Transcript Counts
IFNγ 50 ng/mL
TNFα 25 ng/mL
I
Normalized CXCL10 Transcript Counts
IFNγ 50 ng/mL
TNFα 25 ng/mL
J
Normalized CXCL11 Transcript Counts
IFNγ 50 ng/mL
TNFα 25 ng/mL

Figure S3 (related to Figure 3): Synergistic induction of proinflammatory genes is not dependent on saturation of IFNγ or TNFα signaling

(A) Heatmaps of *IL32*, *GUSB*, *NOL7*, *HPRT1*, *CXCL11*, *CXCL10*, *VCAM1*, *CXCL9*, *SOCS3*, and *CX3CL1* expression in response to 12 -oint dose response curves for IFNγ (left) or TNFα (right) (n=1).

(B-J) Bar plots of normalized *CXCL9* (B,E,H), *CXCL10* (C,F,I), and *CXCL11* (D,G,J) transcript counts after stimulation with 50 ng/mL IFNγ + TNFα 0.024 (B-D), 50 ng/mL IFNγ + TNFα 0.39 ng/mL (E-G), and 50 ng/mL IFNγ + TNFα 25 ng/mL (H-J). Bars indicate mean ± SD (n=4 per condition). One-way ANOVAs were performed followed by Tukey's post-hoc analysis to determine statistical differences between treatment groups. ** $p \leq 0.001$ **** $p \leq 0.0001$

Figure S4

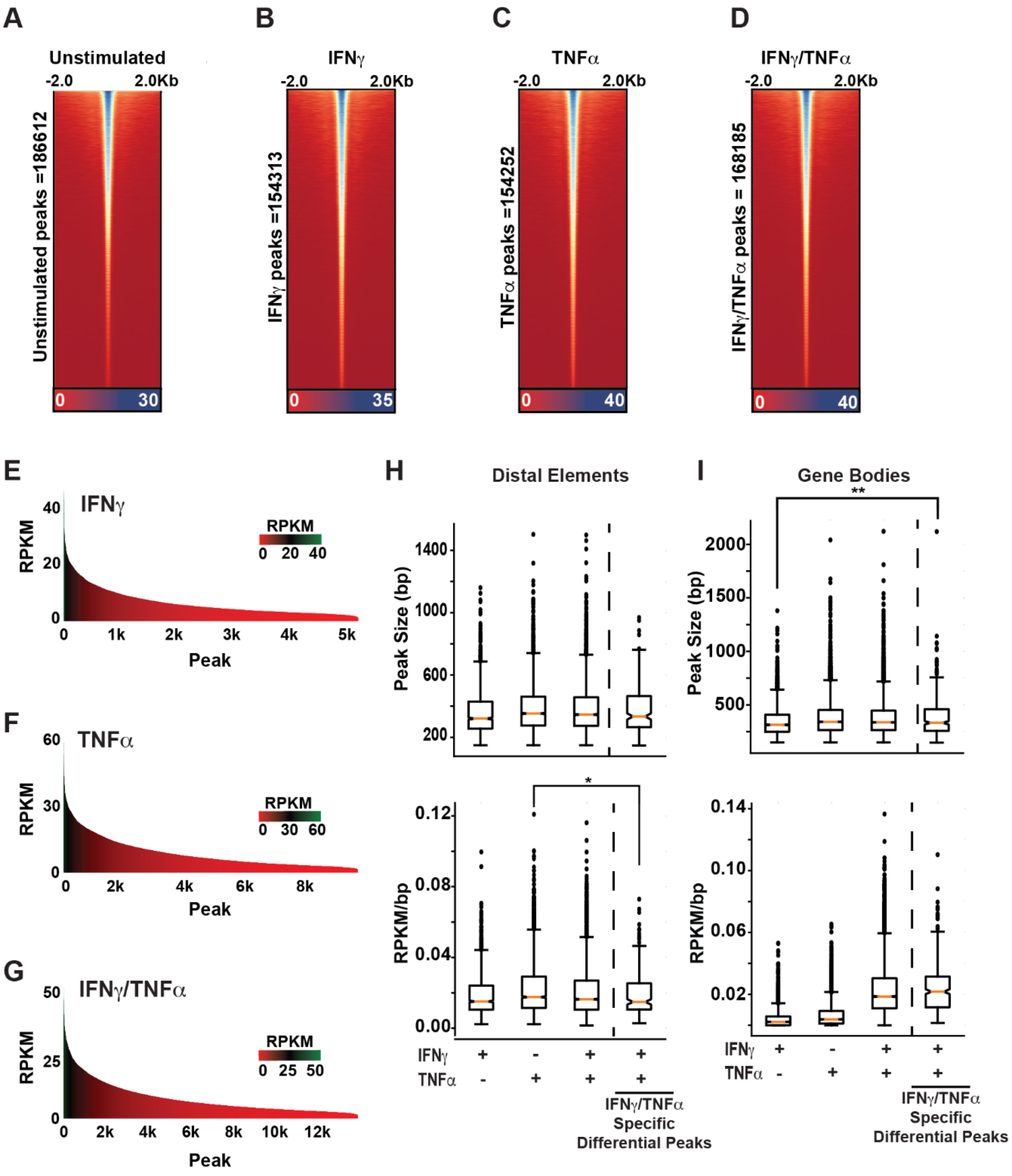

A
Unstimulated
-2.0
2.0Kb
Unstimulated peaks =186612
0
30
B
IFNγ
-2.0
2.0Kb
IFNγ peaks =154313
0
35
C
TNFα
-2.0
2.0Kb
TNFα peaks =154252
0
40
D
IFNγ/TNFα
-2.0
2.0Kb
IFNγ/TNFα peaks = 168185
0
40
E
IFNγ
RPKM
0 20 40
Peak
F
TNFα
RPKM
0 30 60
Peak
G
IFNγ/TNFα
RPKM
0 25 50
Peak
H
Distal Elements
Peak Size (bp)
RPKM/bp
I
Gene Bodies
Peak Size (bp)
RPKM/bp
IFNγ
TNFα
IFNγ/TNFα
Specific
Differential Peaks

Figure S4 (related to Figure 5): Dual cytokine stimulation results in the formation of *de novo* and enhanced accessible elements at the *CXCL-9,-10,11* locus

(A-D) Signal alignment heatmaps for individual ATAC-seq samples.

(E-G) Waterfall plots of RPKM for peaks differential in IFNγ vs unstimulated (E), TNFα vs. unstimulated (F) and IFNγ/TNFα vs. unstimulated (G).

(H, I) Box plots of peak length (top) and normalized signal (bottom) in distal elements (H) and gene bodies (I). Kruskal-Wallis tests were performed followed by pairwise Wilcoxon tests to determine statistical significance. * $p \leq 0.05$ ** $p \leq 0.001$

Figure S5

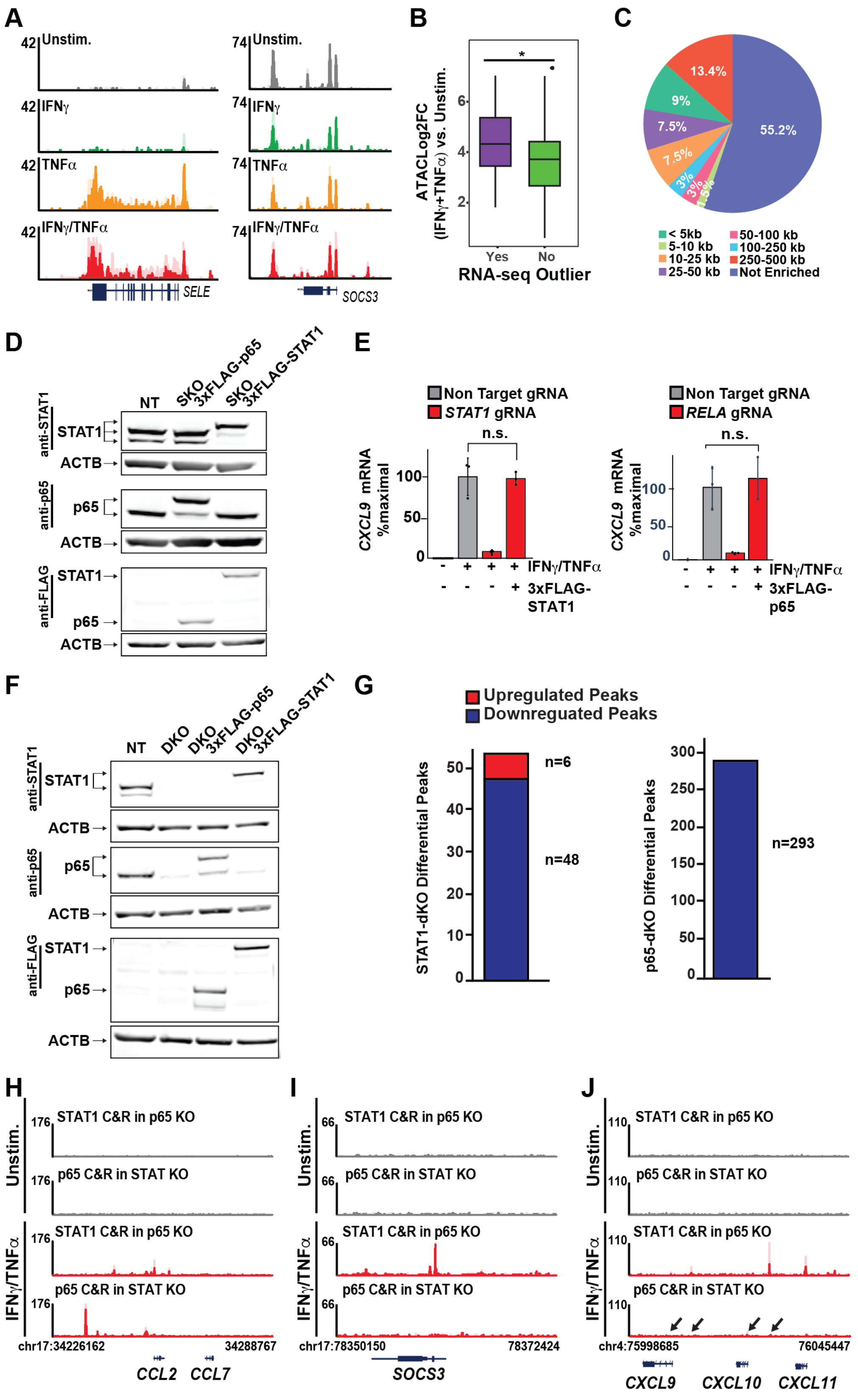

A
Unstim.
IFNγ
TNFα
IFNγ/TNFα
SELE
SOCS3
B
ATACLog2FC (IFNγ+TNFα) vs. Unstim.
Yes
No
RNA-seq Outlier
C
13.4%
9%
7.5%
7.5%
3%
3%
55.2%
< 5kb
5-10 kb
10-25 kb
25-50 kb
50-100 kb
100-250 kb
250-500 kb
Not Enriched
D
NT
SKO 3xFLAG-p65
SKO 3xFLAG-STAT1
anti-STAT1
anti-p65
anti-FLAG
STAT1
p65
ACTB
E
Non Target gRNA
STAT1 gRNA
RELA gRNA
n.s.
CXCL9 mRNA %maximal
IFNγ/TNFα
3xFLAG-STAT1
3xFLAG-p65
F
NT
DKO
DKO 3xFLAG-p65
DKO 3xFLAG-STAT1
G
Upregulated Peaks
Downreguated Peaks
STAT1-dKO Differential Peaks
p65-dKO Differential Peaks
n=6
n=48
n=293
H
I
J
Unstim.
IFNγ/TNFα
STAT1 C&R in p65 KO
p65 C&R in STAT KO
chr17:34226162
34288767
CCL2
CCL7
chr17:78350150
78372424
SOCS3
chr4:75998685
76045447
CXCL9
CXCL10
CXCL11

Figure S5 (related to Figure 5): Dual cytokine stimulation results in the formation of accessible elements and recruitment of STAT1 and p65 at the *CXCL-9*,*-10*, and *-11* locus

(A) Browser tracks of SELE and SOCS3 in cytokine treated HAECs.

(B) Box plot of ATAC Fold Change (IFNγ+TNFα vs. unstimulated) on y-axis vs. RNA-seq outlier status on x-axis. Wilcoxon test was performed followed to determine statistical differences between groups. *p-value < 0.05

(C) Pie chart of percent of outlier genes (65 total) identified in Figure 1 that have an ATAC SSAE site within specified distance to the transcriptional start site of the gene. If a gene had more than one SSAE occurrence, the SSAE closest to the gene was chosen for purposes of counting the total.

(D) Immunoblots of p65 and STAT1 in single KO cells reconstituted with 3xFLAG-p65 or 3x-FLAG-STAT1. For each blot, βactin served as a loading control.

(E) Bar plots of RT-qPCR showing chemokine gene induction in STAT1 KO cells or FLAG-reconstituted p65. Results displayed as percent of maximal induction. Statistical difference tested by one-way ANOVA. n.s. indicates not significant.

(F) Immunoblot of p65 and STAT1 expression in double KO cells after reconstitution with 3xFLAG-p65 or 3xFLAG-STAT1.

(G) Stacked Bar plots of differential peaks for TFs for knockout of STAT1 (left) or RELA (right) TF.

(H-J) Gene tracks of SELE (H), SOCS3 (I) or CXCL locus (J) in double KO cells with p65 or STAT1 reconstitution. Black tracks = unstimulated cells Red tracks = (+) IFNγ/TNFα. C&R = CUT&RUN. Arrows indicate sites where p65 binding has been lost in *STAT1* KO.

Figure S6

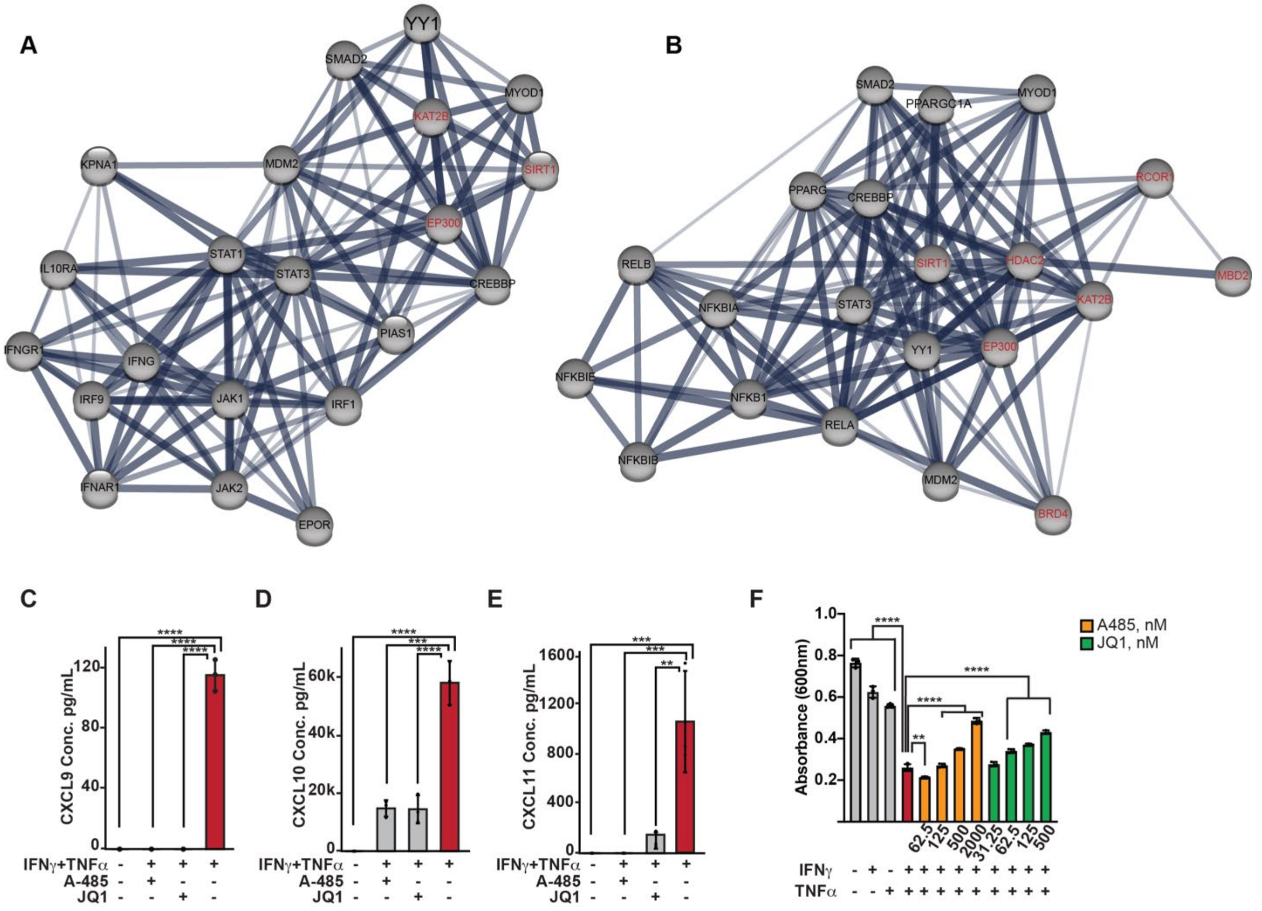

A
B
C
D
E
F
YY1
SMAD2
MYOD1
KAT2B
KPNA1
MDM2
SIRT1
EP300
IL10RA
STAT1
STAT3
CREBBP
PIAS1
IFNGR1
IFNG
IRF9
JAK1
IRF1
IFNAR1
JAK2
EPOR
PPARGC1A
RCOR1
PPARG
RELB
HDAC2
MBD2
NFKBIA
NFKBIE
NFKB1
RELA
BRD4
CXCL9 Conc. pg/mL
CXCL10 Conc. pg/mL
CXCL11 Conc. pg/mL
Absorbance (600nm)
IFNγ+TNFα
A-485
JQ1
IFNγ
TNFα
A485, nM
JQ1, nM
62.5
125
500
2000
31.25

Figure S6 (related to Figure 6): Synergistically induced genes harbor transcriptional dependencies on p300/CBP and BET bromodomain-containing proteins

(A-B) STRING interaction network for STAT1 (A) and RELA/p65 (B) with 1st and 2nd shells set to 10 interactors. Co-activators and proteins involved in histone modifications are highlighted in red.

(C-E) Bar plots of secreted CXCL9 (D), CXCL10 (E), CXCL11 (F) in HAECs treated with dual cytokines and DMSO, A-485 (2000 nM), or JQ1 (500 nM) for 8 hours (n=3 per condition). Data is reported as mean ± SD. One-way ANOVA followed by a Dunnett's test was performed to determine statistically significant changes in chemokines. **$p < 0.01$, ***$p < 0.001$, **** $p < 0.0001$

(F) Bar plot of absorbance at 600 nm as an indicator of cell viability as measured by an MTT assay in HAECs treated co-stimulated with IFNγ/TNFα and various concentrations of A-485 or JQ1. Bars indicate mean ± SD (n=3 per condition). A one-way ANOVA followed by Dunnett's test was performed to assess statistical significance. **$p < 0.01$, ***$p < 0.001$, **** $p < 0.0001$.

Figure S7

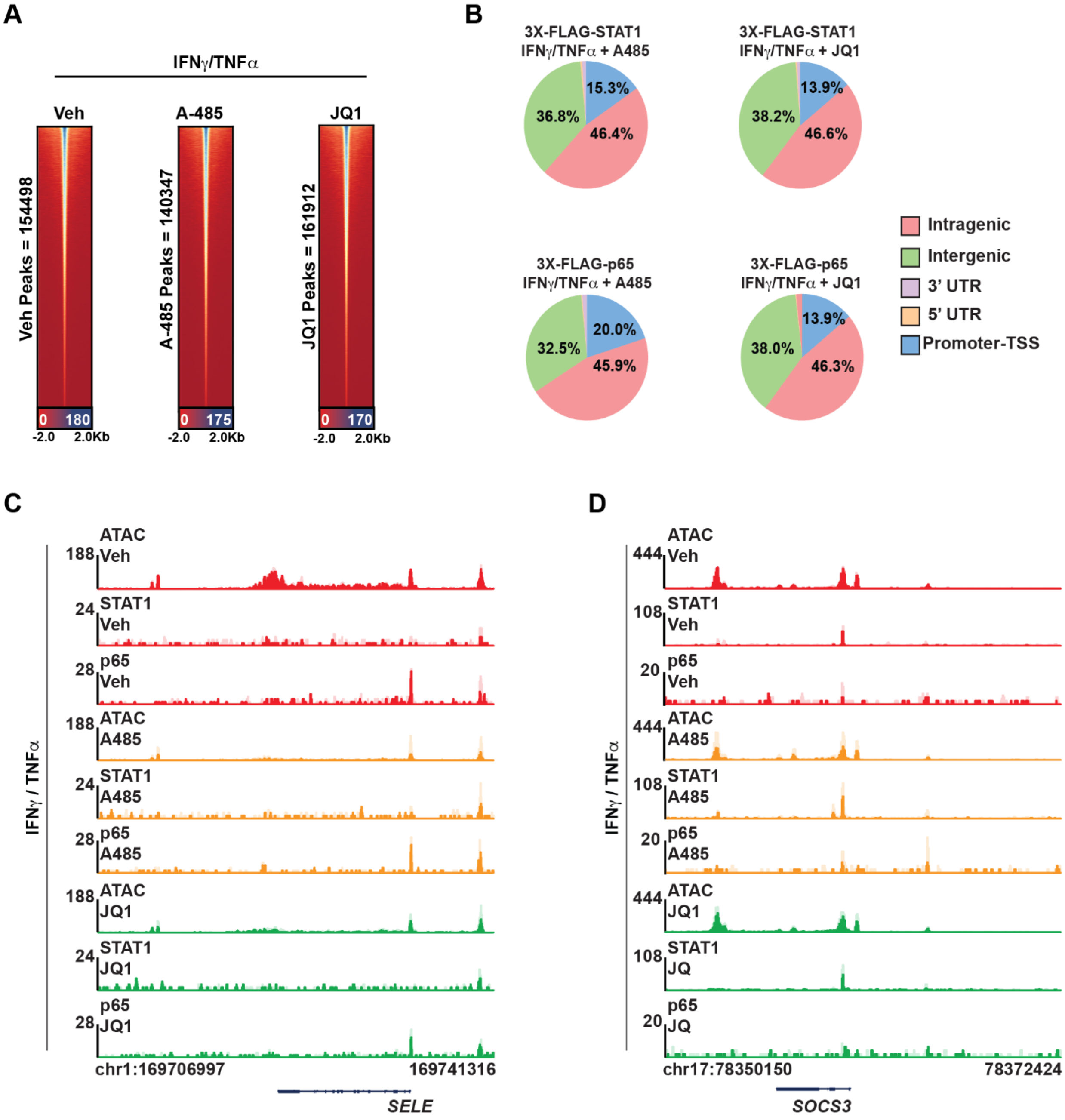

A
IFNγ/TNFα
Veh
A-485
JQ1
Veh Peaks = 154498
A-485 Peaks = 140347
JQ1 Peaks = 161912
0 180
0 175
0 170
-2.0 2.0Kb
B
3X-FLAG-STAT1 IFNγ/TNFα + A485
3X-FLAG-STAT1 IFNγ/TNFα + JQ1
3X-FLAG-p65 IFNγ/TNFα + A485
3X-FLAG-p65 IFNγ/TNFα + JQ1
15.3%
36.8%
46.4%
13.9%
38.2%
46.6%
20.0%
32.5%
45.9%
13.9%
38.0%
46.3%
Intragenic
Intergenic
3' UTR
5' UTR
Promoter-TSS
C
IFNγ / TNFα
188 ATAC Veh
24 STAT1 Veh
28 p65 Veh
188 ATAC A485
24 STAT1 A485
28 p65 A485
188 ATAC JQ1
24 STAT1 JQ1
28 p65 JQ1
chr1:169706997
169741316
SELE
D
IFNγ / TNFα
444 ATAC Veh
108 STAT1 Veh
20 p65 Veh
444 ATAC A485
108 STAT1 A485
20 p65 A485
444 ATAC JQ1
108 STAT1 JQ
20 p65 JQ
chr17:78350150
78372424
SOCS3

Figure S7: (related to Figure 7): p300/CBP or BET bromodomain inhibition does not alter chromatin accessibility or p65 and STAT1 recruitment to chromatin

(A) Heatmap alignment plots of ATAC-seq with individual inhibitors. All samples were treated with dual cytokines.

(B) Venn diagrams of genomic distribution of STAT1 and p65 in CUT&RUN in cytokine treated cells with and without A485 (2 μM) or JQ1 (500 nM).

(C-D) Gene tracks of ATAC-seq along with STAT1 and p65 CUT&RUN at *SELE* (C) and *SOCS3* (D) loci.

Table S1: (related to Figure 2): Fold-Change Values for WT, p65 KO and STAT1 KO HAECs in Response to IFNγ/TNFα Dual Stimulation

**Fold Change By Gene**	WT (STAT+/p65+)	p65 KO	STAT KO
CXCL9	2041	101	39
CXCL10	215329	1183	5935
CXCL11	205	31	6.6
